# Borosulfates Hosting Se_4_^2+^ Ions—Crystal Structures, Chemical Bonding, Optical Spectroscopy, and Magnetism

**DOI:** 10.1002/anie.4300417

**Published:** 2026-09-19

**Authors:** Erich Turgunbajew, Marios Symeonidis, Jörn Bruns, Sara N. Özbay, Jan Langwald, Mathias S. Wickleder, Robert E. Dinnebier, Linda S. Reitz, Richard Dronskowski, Lars Schumacher, Rainer Pöttgen, Henning A. Höppe

**Affiliations:** ^1^ Lehrstuhl für Festkörperchemie Universität Augsburg Augsburg Germany; ^2^ Institut für Chemie Technische Universität Berlin Berlin Germany; ^3^ Institut für Anorganische und Materialchemie Universität zu Köln Köln Germany; ^4^ Röntgenographie, Max‐Planck‐Institut für Festkörperforschung Stuttgart Germany; ^5^ Institut für Anorganische Chemie RWTH Aachen University Aachen Germany; ^6^ Institut für Anorganische und Analytische Chemie Universität Münster Münster Germany

**Keywords:** borosulfates, powder reflectance spectroscopy, selenium, thermal analysis, weakly coordinating anion

## Abstract

Despite consistent growth in the number of compounds known to host homo‐ and hetero polycations of group 16 elements, only two, Se_4_[HS_2_O_7_]_2_ and Se_4_[S_4_O_13_], provided an exclusively oxidic coordination environment, the former being the first structural characterization of the Se_4_^2+^ cation in 1968. With the first selenium borosulfates Se_4_[B(S_2_O_7_)_2_]_2_(SO_3_), Se_4_[B(S_2_O_7_)_2_]_2_, Se_4_[B_2_(SO_4_)_2_(S_2_O_7_)_2_]‐I, Se_4_[B_2_(SO_4_)_2_(S_2_O_7_)_2_]‐II, and Se_4_[B_2_(SO_4_)_4_] we extended this list by five compounds. Aside from Se_4_[HS_2_O_7_]_2_, Se_4_[S_4_O_13_] was synthesized but never structurally characterized. In this contribution we also evaluate the crystal structure of the tetrasulfate Se_4_[S_4_O_13_]. All compounds are synthesized under solvothermal conditions. Powder reflectance spectra of the selenium borosulfates are in accordance with the yellow body color of the compounds. Magnetic measurements confirm the diamagnetic character of the Se_4_^2+^ ions, in agreement with the calculations. Temperature‐programmed powder X‐ray diffraction reveals that the borosulfates of the study form sequentially with increasing decomposition temperature, enabling their selective synthesis. Furthermore, we performed infrared spectroscopy, Rietveld refinements and thermal analyses.

## Graphical abstract

Apart from (transition) metal (oxo)halides only few hosts stabilize chalcogenide cations in purely oxoanionic coordination environment. Herein, we report on five borosulfates containing the Se_4_^2+^ ion and characterize the compounds using X‐ray diffraction, diffuse reflectance spectroscopy, density functional theory (DFT) calculations along with magnetic measurements and thermal analysis. Moreover, the crystal structure of Se_4_[S_4_O_13_] is reported.

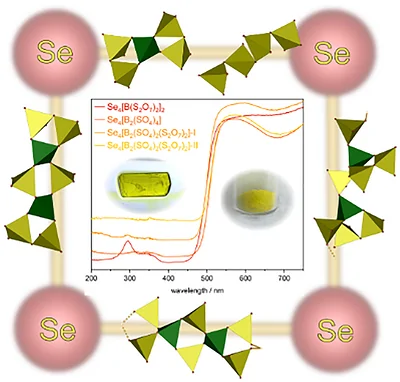

## 1 Introduction

For a long time, strongly acidic and colorful solutions of chalcogens have fascinated chemists. As early as 1783, Müller von Reichenstein reported a solution showing an “unvergleichlich schöne gesättigte Karmoisinfarbe” – an incomparably beautiful, saturated crimson red color. This solution resulted from dissol‐ving a metal, that he had extracted from a gold ore in sulfuric acid [1]. At that time, the element was still unknown and it took another 15 years until Klaproth confirmed the discovery of this new element which he then named tellurium [2]. In the early 19th century, Buchholz and Berzelius made similar observations when they dissolved the chalcogenides sulfur and selenium in sulfuric and selenic acid, respectively, yielding blue and yellow solutions [3, 4, 5]. The origin of the colored solutions remained mysterious for the following 150 years, despite numerous hypotheses regarding their nature [6, 7, 8, 9, 10, 11, 12]. Furthermore, the oxidation states of the chromophores were unknown, and the question whether the chromophores were radicals was controversially debated [10, 13, 14, 15, 16, 17]. This topic remained subject to discussion until Bjerrum et al. and Gillespie et al. independently determined the color origin for tellurium and selenium in 1968. While Gillespie et al. investigated the origin of the colored species in highly acidic media like HSO_3_F, H_2_S_2_O_7_ and H_2_SO_4_, Bjerrum et al. chose the Lewis acidic molten salt AlCl_3_‐NaCl. Both proposed that the colors originate from polyatomic Se_4_^2+^, Se_8_^2+^, and Te_4_^2+^ cations; still, their topology was unknown [18, 19]. In the same year, Gillespie et al. published the first compounds containing Se_4_^2+^ cations in Brønsted acidic media including Se_4_[S_4_O_13_] and Se_4_[HS_2_O_7_]_2_. Later the year, the latter was subject to the first single crystal X‐ray diffraction study, revealing the square planar shape of the cation [20, 21, 22]. Shortly thereafter, also the first tellurium and sulfur polycations were isolated in Te_x_[AlCl_4_] [23] and S_8_[AsF_6_]_2_ [24] by Corbett and Gillespie. Over the following years, more complex chalcogen homo– and heteropolyatomic cations also mixing elements of main‐groups V through VII, were isolated in solids. In this context, the contributions to this topic by the group of Johannes Beck ought be highlighted [25, 26, 27, 28]. Intriguingly, while these cations were initially found in Brønsted acidic media, the stabilization in the subsequent studies almost exclusively occurred in fluorido‐antimonates and ‐arsenates and other halidometallates [29, 30]. Until this year, Se_4_[HS_2_O_7_]_2_ and Se_4_[S_4_O_13_] remained the only purely oxoanionic compounds known to stabilise these polycations, whereby a recent contribution by Wickleder et al. brought a deeper insight into the stabilizing potential of oxoanions towards the *Ch*_4_^2+^ (*Ch* = Se, Te) cations as well as Te_6_^4+^ [31].

The conjugated bases of strong Brønsted and Lewis acids exhibit weak coordination behavior that appears to be a precondition for the stabilization of such cations. In 1971, Gillespie determined the Hammett acidity function for several selected acidic systems including the H_2_SO_4_/HB(HSO_4_)_4_ system. Hence, the *H*_0_ values were determined to be approximately –13, on par with HSO_3_F, characterizing borosulfonic acid as superacidic [32]. Accordingly, the deprotonated anion [B(SO_4_)_4_]^5–^ provides a weak coordination. Today, this anion is regarded as the typical basic building unit of borosulfates, a comparably new material class with their first crystallographically characterized representative K_5_[B(SO_4_)_4_] published in 2012 [33]. Borosulfates typically constitute of alternating sulfate and borate tetrahedra sharing common vertices to produce zero‐, as in Li_5_[B(SO_4_)_4_] [34], one‐, as in Ag[B(SO_4_)_2_] [35], two‐ as in Cd[B_2_(SO_4_)_4_] [36], and three‐dimensional anions like in Li[B(SO_4_)_2_] [37]. The weak coordination strength of borosulfates was reported several times. For instance, diffuse reflectance spectra of α‐*M*_4_[B_2_O(SO_4_)_6_] (*M* = Co, Ni) revealed small Racah parameters and ligand field splitting compared to their respective sulfates and chlorides [38]. Additionally, luminescence spectroscopy of Ce_2_[B_2_(SO_4_)_6_] unveiled emissions deep in the UV regime comparable to fluorides [39]. Recently, ^121^Sb Mössbauer spectra of Sb*A*[B(SO_4_)_2_]_4_ (*A* = K, Rb, Cs, NH_4_) revealed the most negative isomer shift to date of almost –22 mm·s^−1^, confirming the anion´s weak coordination strength [40]. Additionally, in I_4_[B(S_2_O_7_)_2_]_2_ the structurally related homopolycation I_4_^2+^ was stabilized, previously only reported in weakly coordinating anions like fluoridoantimonates [41].

The research on these homo‐ and heteropolycations of the elements in the main groups V, VI, and VII mainly focused on the discovery, their synthesis, stabilization and structural analysis of new polycations in metal halides, and their theoretical treatment. If any further investigations on the properties of the cations were carried out, these were typically performed in solution. Herein, we present five borosulfates and a polysulfate containing Se_4_^2+^ ions in a purely oxoanionic coordination environment. We elucidate their crystal structures and, for the first time, present UV–vis spectra of Se_4_^2+^ cations in solids. Furthermore, we performed thermogravimetric analyses (TGA) and temperature programmed powder X‐ray diffraction (TPPXRD) experiments highlighting the structural relationship between all presented borosulfates. These results are complemented by DFT calculations, infrared spectroscopy (IR) and magnetic measurements.

## 2 Results and Discussion

### 2.1 Crystal Structures Se_4_[S_4_O_13_]

Elemental selenium dissolved in oleum (65% SO_3_) at room temperature leads to the formation of selenium hydrogendisulfate Se_4_[HS_2_O_7_]_2_, already shown by Gillespie et al. in the 1960´s [20]. The Wickleder group focuses on the synthesis of polysulfates and found that, while SO_3_‐rich oleum (65% SO_3_) typically only results in disulfates, using neat SO_3_ enhances the formation of polysulfates with higher condensation degrees, reaching up to hexasulfates like (NH_4_)_2_[S_6_O_19_] and Rb_2_[S_6_O_19_] which constitute the longest polysulfates known till date [42]. With Se_4_[S_4_O_13_] we introduce a new representative comprising the still quite rare tetrasulfate [S_4_O_13_]^2–^ anion (Figure 1a). The latter was first reported only a decade ago along with the synthesis of (NO_2_)_2_[S_4_O_13_] [43]. Even though the composition of Se_4_[S_4_O_13_] was already determined accurately by a gravimetric analysis in 1968 by Gillespie et al., its structure remained unknown – until now [20]. Se_4_[S_4_O_13_] crystallizes in the orthorhombic space group *P*2_1_2_1_2_1_ (no. 19) with eight formula units per unit cell – and due to the weak coordination strength of the polysulfate intrinsically as inversion twin, though. The structure consists of two crystallographically independent tetrasulfate anions arranged in a strongly distorted fcc‐like manner where the Se_4_^2+^ rings fill the octahedral voids and are coordinated by seven tetrasulfate anions each (Figure 1). The latter are present in a zig zag arrangement with a dihedral angle close to 180°. This is in line with the observed sulfate chain conformation within the halogen tetrasulfates (IX_2_)_2_[S_4_O_13_] (X = Br, I) recently reported by Wickleder et al. [44]. Contrarily, group I and II tetrasulfates show (except the hydrogenium‐*bis*‐tetrasulfate anion [H(S_4_O_13_)_2_]^3–^), a predominantly horseshoe‐type arrangement [45]. A direct comparison between both conformations is shown in the supplement (Figure S1). The bond lengths in the anion are comparable to the lithium compound Li_2_[S_4_O_13_]: terminal S–O bonds range between 141 and 144 pm, whereas the S–O–S bridges exhibit a bond length asymmetry, that is alternating long and short bonds between 154 and 175 pm (Table S7), which we also analyzed previously. The terminal S–O bonds coordinating towards the Se_4_^2+^ cation show the largest bond extension and the strongest distortion of the sulfur centre towards a tetrahedral geometry, due to strong non‐covalent interactions with the cation. Notably, the observed terminal bond lengths and sulphate geometries indicate that the Lewis acidity of the Se_4_^2+^ cation is in the same order of magnitude as for the Li^+^ cation.

**FIGURE 1:**
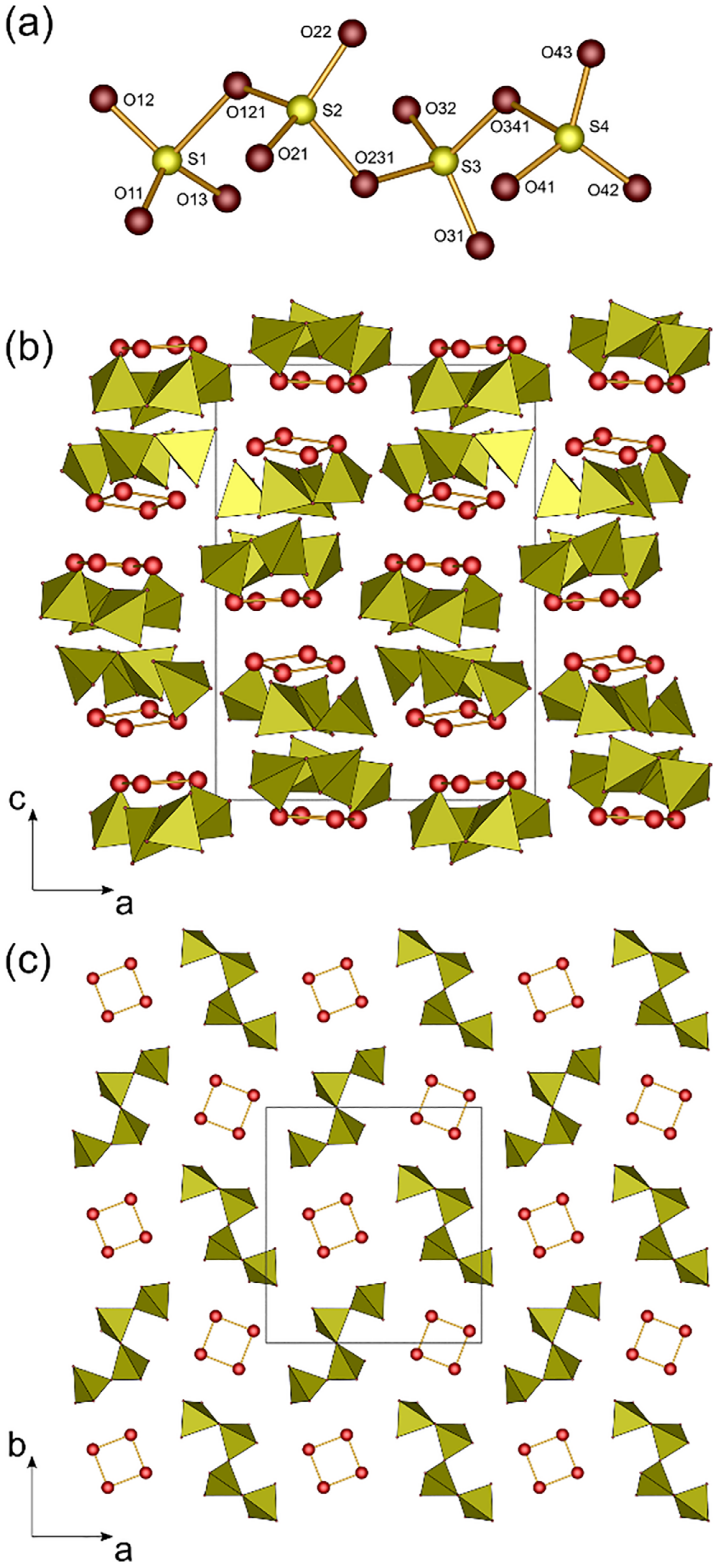
(a) S_4_O_13_^2–^ unit of Se_4_[S_4_O_13_]. (b, c) Unit cell of Se_4_[S_4_O_13_] along different directions. For a better understanding of the structural arrangement in (b) only a single layer is displayed.

### 2.2 Se_4_[B(S_2_O_7_)_2_]_2_(SO_3_)

While the reactions in neat SO_3_ and oleum (65% SO_3_) at moderate temperatures typically lead to polysulfates as discussed above, adding boron oxide to such reactions typically yields unconventional borosulfates featuring (S_2_O_7_)^2–^ units with S–O–S bridges. Consequently, in this study four such borosulfates were prepared. The first, Se_4_[B(S_2_O_7_)_2_]_2_(SO_3_), crystallizes in the monoclinic space group *C*2/*c* (no. 15) with four formula units per unit cell (Figure 2). This structure can be derived from the AlB_2_ structure type where the [B(S_2_O_7_)_2_]^2–^ units resemble the graphene‐like boron layers and the selenium cations occupy all hexagonal prismatic sites (Figure S2). Subsequently, each Se_4_^2+^ cation is coordinated by twelve [B(S_2_O_7_)_2_]^2–^ anions and two additional SO_3_ molecules, located between adjacent Se_4_^2+^ cations. The [B(S_2_O_7_)_2_]^–^ anion is well known in literature occurring with simple mono‐ and divalent cations like in *A*[B(S_2_O_7_)_2_] (*A* = Li, Na, K, NH_4_, Ag) [34, 37, 46, 47] and Ba[B(S_2_O_7_)_2_]_2_ [48] but also more exotic oligocations like in the aforementioned I_4_[B(S_2_O_7_)_2_]_2_ [41] and [Au_2_Cl_4_][B(S_2_O_7_)_2_](SO_3_) [49]. Within the anion we deduced typical bond lengths, comparable to the previously mentioned compounds featuring *bis*–disulfatoborate anions (Table S5).

**FIGURE 2:**
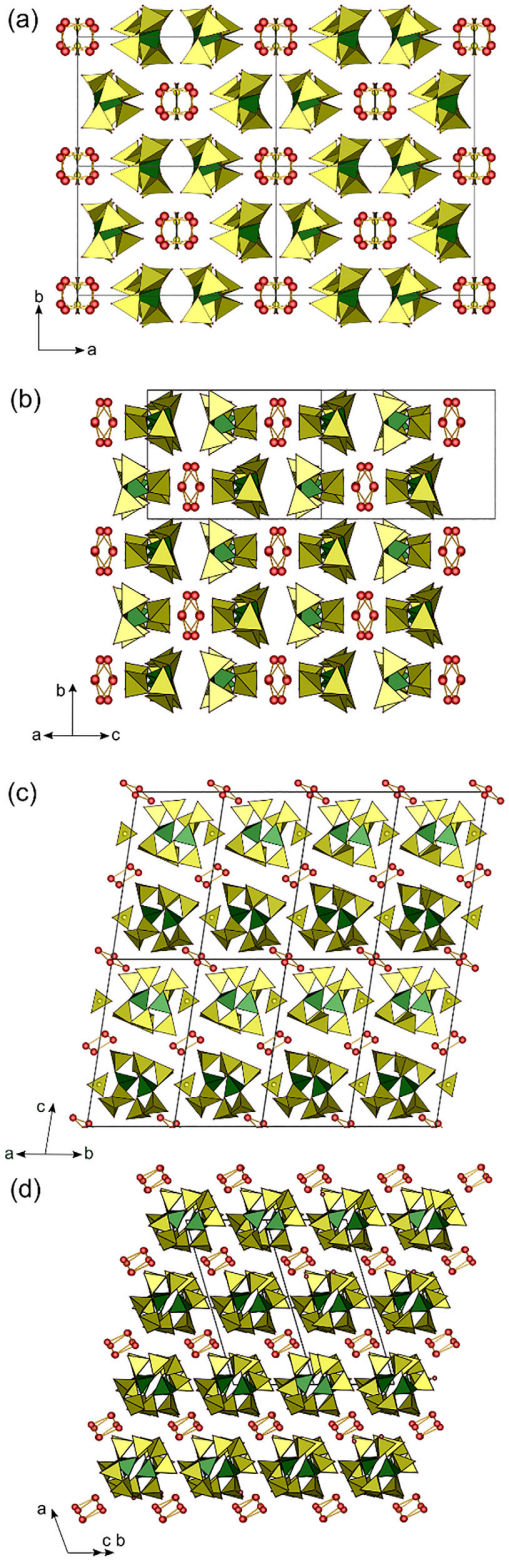
Comparison of the structures of Se_4_[B(S_2_O_7_)_2_]_2_(SO_3_) (a + c) and Se_4_[B(S_2_O_7_)_2_]_2_ (b + d) along two different viewing directions.

### 2.3 Se_4_[B(S_2_O_7_)_2_]_2_

The SO_3_ molecule in Se_4_[B(S_2_O_7_)_2_]_2_(SO_3_) is bound quite weakly and can be readily removed to give Se_4_[B(S_2_O_7_)_2_]_2_. By storing the SO_3_‐rich modification for a few days in a glovebox, the removal already occurs at room temperature and is completed after a few weeks. The Se_4_[B(S_2_O_7_)_2_]_2_ structure was solved from powder X‐ray diffraction data (details in the Supporting Information) and crystallizes in the monoclinic space group *C*2/c (no. 15) with four formula units per unit cell. Despite a reorientation of the molecular ions, the coordination number for the Se_4_^2+^ cations as well as the structural motifs with their AlB_2_‐type arrangement are retained, where the Se_4_‐units occupy the Al and [B(S_2_O_7_)_2_]^–^ anions replace the B positions. As expected, the loss of SO_3_ results in a shrunk unit cell (Figures 2b,d, Table S2). All bond lengths remain in the expected range (Table S5).

### 2.4 Se_4_[B_2_(SO_4_)_2_(S_2_O_7_)_2_]‐I

Two polymorphs fulfil the composition Se_4_[B_2_(SO_4_)_2_(S_2_O_7_)_2_]. Se_4_[B_2_(SO_4_)_2_(S_2_O_7_)_2_]‐I adopts space group *P*2_1_/*c* (no. 14) with two formula units per unit cell and comprises Se_4_^2+^ cations and zero‐dimensional [B_2_(SO_4_)_2_(S_2_O_7_)_2_]^2–^ units. These units constitute a new anion topology for borosulfates; it can be considered as a modification of the cyclic anion found in the rare‐earth borosulfate series *R*_2_[B_2_(SO_4_)_6_] (*R* = Y, La–Nd, Sm–Lu) [39] where two of the four terminal (SO_4_) units condense to two chelating (S_2_O_7_) units. Hence, the two boron atoms are bridged by two (SO_4_) groups forming *vierer* rings and additionally are coordinated by a chelating (S_2_O_7_) unit each. Consequently, according to Liebau´s nomenclature the anion is defined as a loop‐branched *vierer* single ring [50]. All observed bond lengths agree with the sum of the ionic radii and other borosulfates featuring S–O–S bridges (Table S5). As illustrated in Figure 3, viewing along the *a*‐direction, the Se_4_^2+^ cations as well as the [B_2_(SO_4_)_2_(S_2_O_7_)_2_]^2–^ anions, form close packed layers even though these are not packed alike hcp where the spheres of layer B are situated above the triangles of spheres of layer A; here the Se_4_^2+^ cations lie above and below parallelograms formed by [B_2_(SO_4_)_2_(S_2_O_7_)_2_]^2–^ anions leading to an eightfold coordination by the latter.

**FIGURE 3:**
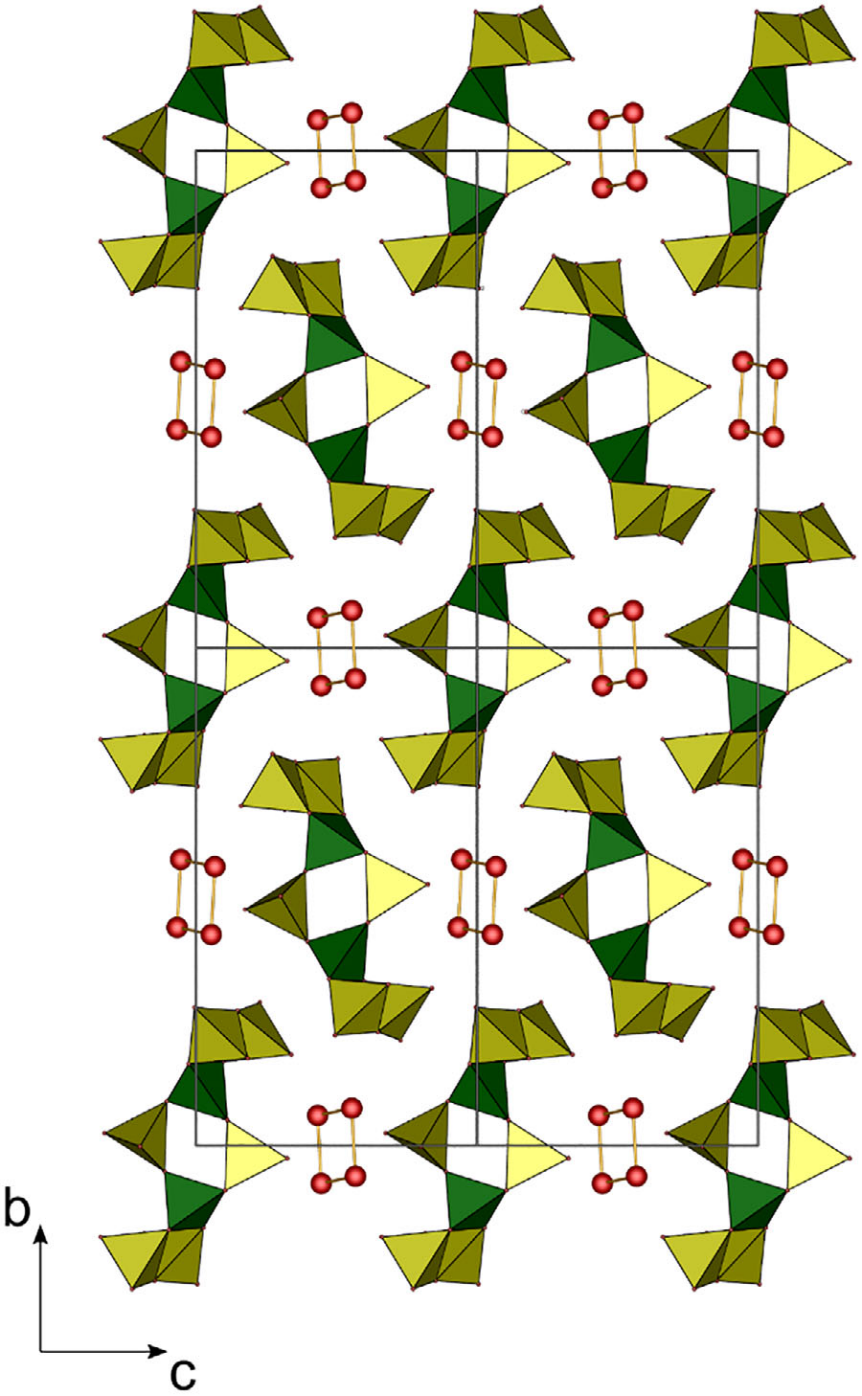
2×2 unit cells of Se_4_[B_2_(SO_4_)_2_(S_2_O_7_)_2_]–I viewed along [100].

### 2.5 Se_4_[B_2_(SO_4_)_2_(S_2_O_7_)_2_]‐II

The second polymorph of Se_4_[B_2_(SO_4_)_2_(S_2_O_7_)_2_] crystallizes also in space group type *P*2_1_/*c* (no. 14) as the first one and exhibits four formula units per unit cell. Contrarily, it features a one‐dimensional anion instead, known from *A*[B(SO_4_)(S_2_O_7_)] (*A* = Cs, H) and the recently published Pd[B(SO_4_)(S_2_O_7_)]_2_ (Figure 4a) [46, 51]. Similarly, to the first polymorph, the central boron atoms are coordinated by two (SO_4_) and a chelating (S_2_O_7_) subunit. Compared to the first polymorph, however, where both (SO_4_) units coordinate to the same boron atom to form *vierer* single rings, these rings are opened to coordinate two distinct boron atoms and hence form chains running along the [100] direction (Figure 4b). These chains can be described as loop‐branched *vierer* single chains. The observed bond lengths agree well with other borosulfates (Table S5). Charge compensation is achieved by one crystallographically independent Se_4_^2+^ cation, coordinated by four chains each.

**FIGURE 4:**
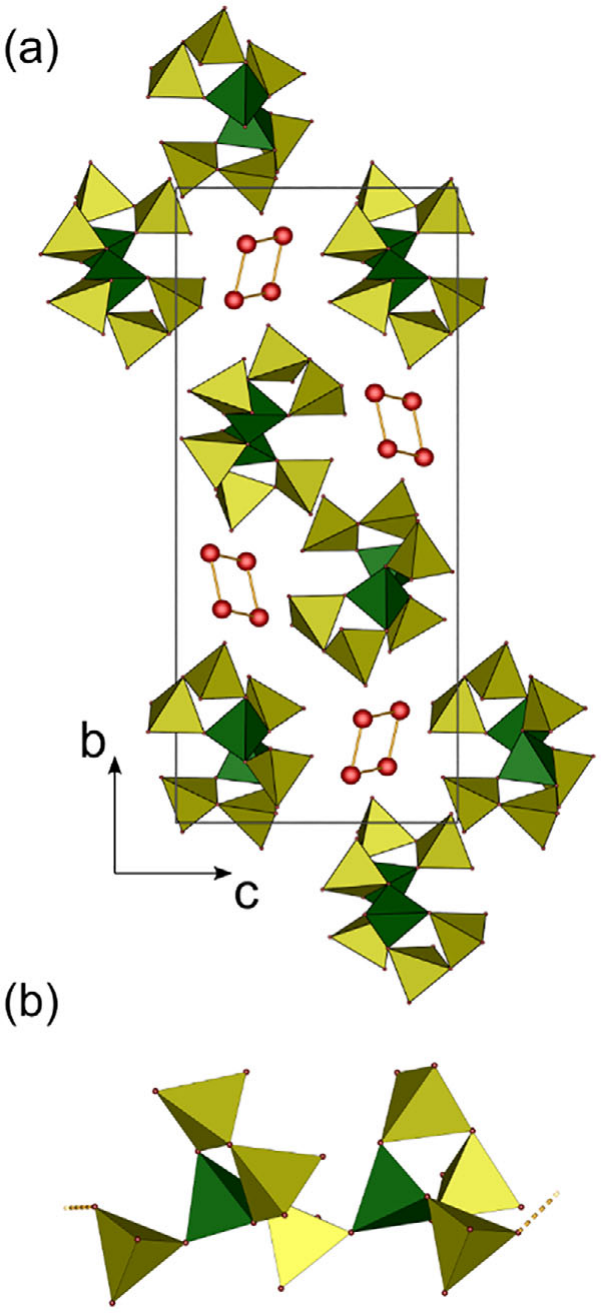
(a) Unit cell viewed along [100] and (b) the fundamental building unit of Se_4_[B_2_(SO_4_)_2_(S_2_O_7_)_2_]‐II.

### 2.6 Se_4_[B_2_(SO_4_)_4_]

Se_4_[B_2_(SO_4_)_4_] crystallizes in space group *C*2/*c* (no. 15) with two formula units per unit cell (Figure 5). Alternating borate and sulfate tetrahedra share common corners and form chains running along [010], analogous to SiS_2_ (Si → B, S → SO_4_). While each borate is connected to four adjacent sulfate tetrahedra each sulfate tetrahedron connects two borate tetrahedra forming *vierer* rings. Accordingly, the anion can be described as loop branched *vierer* single chains exhibiting the Niggli formula _∞_^1^{[B(SO_4_)_4/2_]^–^}. This anion topology is common to borosulfate chemistry and found for example in *A*[B(SO_4_)_2_] (*A* = H_3_O, NH_4_, K, Ag) [34, 35, 46] and *M*[B(SO_4_)_2_] (*M* = Sr, Pb, Ba) [48, 52, 53]. A comparison of these compounds with Se_4_[B_2_(SO_4_)_4_] reveals a clear difference in the arrangement of the chains. It is evident that the chains tilt to accommodate the cations’ size and non‐spherical shape. The selenium rings are stacked along [100] and coordinated by four chains each. All observed bond lengths in the (BO_4_) and (SO_4_) tetrahedra are typical for compounds with this structural motif and are presented in Table S5.

**FIGURE 5:**
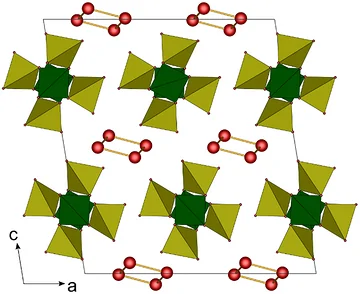
Unit cell of Se_4_[B_2_(SO_4_)_4_] viewed along [010].

The coordination environment of the Se_4_^2+^ cations in all six compounds resembles the specifics of *Ch*_4_^2+^ cations (*Ch* = chalcogen) and is exemplified in Figure 6 for Se_4_[B_2_(SO_4_)_4_] while the remaining ones are depicted in the Figure S3. The shortest contacts are consistently observed to four oxygen atoms bridging the edges of the Se_4_^2+^ square with mean values between 272 and 293 pm. The total coordination number (CN) was confirmed via MAPLE calculations (Madelung Part of Lattice Energy) and ranges between 17 for Se_4_[B_2_(SO_4_)_2_(S_2_O_7_)_2_]‐II and 20 for Se_4_[S_4_O_13_] considering a threshold of 370 pm (Tables S8‐S13) [54, 55, 56, 57, 58]. These high coordination numbers are common for smaller coordinating atoms like oxygen and fluorine and decrease with rising ionic radii in chlorides and bromides. Not only does the size and hence electron density influence the CN but also the coordination strength and the bond lengths within the cation; even the color of the compound correlates with the CN. Based on the limited number of compounds known at the time, Burns et al. concluded that increasing polarizability or, alternatively increasing its charge density, strengthens the cation‐anion interactions leading to a bond elongation. Hence, the energy gap in Se_4_^2+^ between the HOMO and LUMO formed by the selenium p‐orbitals decreases and the color of the compounds shifts from yellow to orange [59, 60]. Table 1 lists the mean Se–Se distances as well as the color of the compounds containing Se_4_^2+^ ions; here, only compounds were included where the color can reasonably be attributed to the Se_4_^2+^ cations. We can confirm the general trend that with increasing Se–Se distance a shift from yellow over orange to red is observed. Albeit this rule does not hold strictly, for example Se_4_[S_4_O_13_] and Se_4_[AlCl_4_]_2_. Although Se_4_[S_4_O_13_] exhibits an average bond length greater than Se_4_[AlCl_4_]_2_, the former is yellow while the latter shows an orange hue (Table 1). The statistical error of these averaged bond lengths is based on the esds of the refinements. Accordingly, the color hue of the compounds is a more reliable indicator for the coordination strength of a host structure than considering the mean Se–Se distances only. Thus, exhibiting a yellow body, the tetrasulfate and borosulfate anions reported in this study can be considered at least as weakly coordinating as the compound class of fluoridoantimonates. Regarding the molecular cations, all Se_4_^2+^ rings within the borosulfates exhibit mean bond lengths between 227.95(6) and 228.53(6) pm ranking approximately between the fluorides and chlorides (Table 1); moreover, the Se_4_^2+^ cations only slightly deviate from *D*_4*h*_ symmetry. Detailed geometrical data are listed in the Table S6. All compounds were synthesised as phase pure crystalline powders (Figures S5–S10).

**FIGURE 6:**
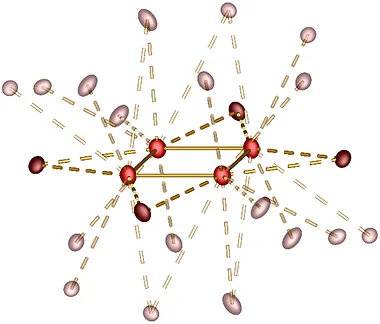
Typical coordination environment of Se_4_^2+^ in borosulfates exemplified by Se_4_[B_2_(SO_4_)_4_]. The oxygen atoms coordinating the edges of the rings are highlighted. Oxygen atoms in dark red; selenium in red. Displacement ellipsoids are set to 70% probability.

**TABLE 1:** Mean Se–Se distances and color appearance of Se_4_^2+^ cations in Se_4_[S_4_O_13_], Se_4_[B(S_2_O_7_)_2_]_2_(SO_3_), Se_4_[B(S_2_O_7_)_2_]_2_, Se_4_[B_2_(SO_4_)_6_], Se_4_[B_2_(SO_4_)_2_(S_2_O_7_)_2_]‐I, and Se_4_[B_2_(SO_4_)_2_(S_2_O_7_)_2_]‐II compared to compounds reported previously

Compound	Mean Se–Se distance / pm	Se‐distance range / pm	Point group	Color appearance	Ref.
Se_4_[Sb_2_F_4_][Sb_2_F_5_][SbF_6_]_5_	226.1(6)	224.7‐227.6	*D*_2*h*_	yellow	[61]
Se_4_[SbF_17_][SbF_6_]_3_	227.2(5)	226.5‐227.5	*D*_2*h*_	yellow	[62]
Se_4_[B_2_(SO_4_)_4_]	227.95(6)	227.9‐228.0	*D*_2*h*_	yellow	this work
Se_4_[B_2_(SO_4_)_2_(S_2_O_7_)_2_]‐II	228.08(8)	227.6‐228.7	*C*_1_	yellow	this work
Se_4_[HS_2_O_7_]_2_	228.3(6)	228.0‐228.6	*D*_2*h*_	orange	[20]
Se_4_[B_2_(SO_4_)_2_(S_2_O_7_)_2_]‐I	228.49(8)	228.1‐228.9	*D*_2*h*_	yellow	this work
Se_4_[B(S_2_O_7_)_2_]_2_(SO_3_)	228.53(6)	228.5‐228.6	*D*_2*h*_	yellow	this work
Se_4_[AlCl_4_]_2_	228.5(2)	228.3‐228.8	*D*_2*h*_	orange	[61]
Se_4_[Bi_6_F_11_][AsF_6_]_9_ · 10 SO_2_	228.8(2)	228.1‐229.5	*D*_2*h*_	yellow	[63]
Se_4_[S_4_O_13_]	228.9(1)	228.5‐229.2	*C*_1_	yellow	this work
Se_4_[S_2_O_7_]	228.57(3)	228.1‐229.1	*C*_1_	orange	[31]
Se_4_[HS_3_O_10_]	228.62(3)	228.5‐228.8	*C_i_*	yellow	[31]
Se_4_[HfCl_6_]	229.18(9)	229.2	*D*_2*h*_	light red	[64]
Se_4_[ZrCl_6_]	229.7(3)	229.2‐230.1	*D*_2*h*_	light red	[64]
Se_4_[Bi_4_Cl_14_]	229.6(1)	229.6	*D*_4*h*_	red	[65]

### 2.7 Chemical Bonding in Se_4_[B_2_(SO_4_)_4_]

To shed some quantum‐chemical light on the bonding situation, especially in the Se_4_‐unit, the crystal structure of Se_4_[B_2_(SO_4_)_4_] was DFT‐optimized and the resulting wavefunctions were projected onto a local atomic‐orbital basis using the LOBSTER suite. This procedure allowed us to calculate a local DOS, precise bond orders and a molecular orbital scheme of the Se_4_‐unit [66]. All computational details are provided in the Supporting Information.

Figure 7a shows the total DOS. The most significant contributions stem from the oxygen's 2s and 2p, the selenium's 4p and sulfur's 3p orbitals. The calculated Löwdin charges (Figure 7b) are provided next to the corresponding atoms in the Se_4_[B_2_(SO_4_)_4_] unit. The integrated charge for the Se_4_‐unit is +1.64 which is close to the ionic‐limit value of +2. Both, the sulfur and boron atoms are coordinated tetrahedrally by oxygen. The Löwdin charge on the oxygen atoms surrounding boron amounts to −0.70. The terminal oxygen atoms of the sulfate tetrahedra show a Löwdin charge of −0.90.

**FIGURE 7:**
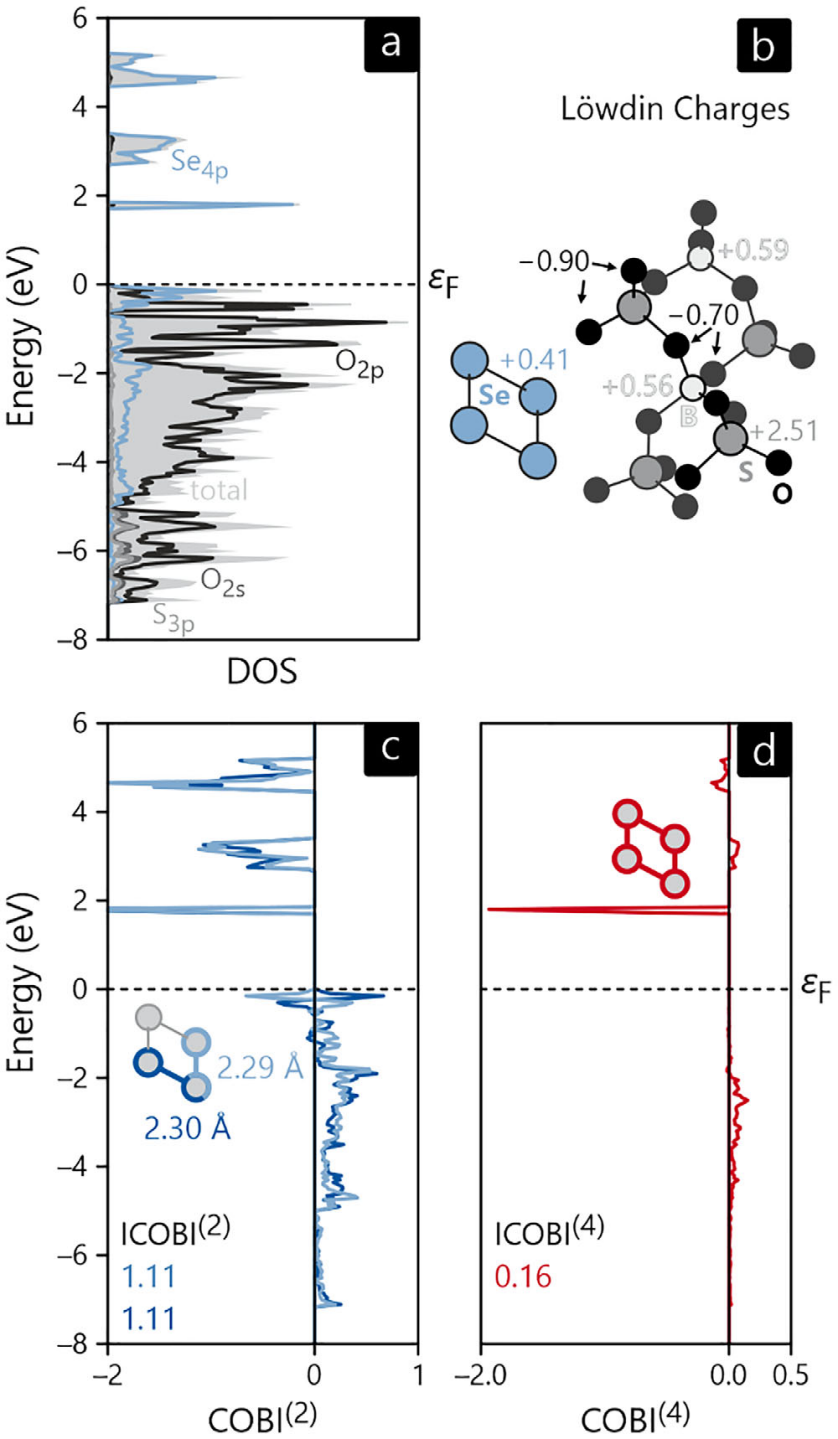
(a) Total‐DOS shown in light grey with the most signi‐ficant contribution from oxygen's 2s and 2p (highlighted in dark grey and black) selenium's 4p (blue) and sulfur's 3p (grey) orbitals. (b) Schematic depiction of a Se_4_[B_2_(SO_4_)_4_] unit with the charges for each species. Plots of the Crystal Orbital Bond Index for (c) two and (d) four centres are provided with their corresponding integrated (ICOBI) values.

To better understand the bonding of the Se_4_‐unit, the two‐centre as well as the four‐centre Crystal Orbital Bond Indices (COBI) were calculated [67]. The COBI as well as the corresponding integrated values (ICOBI) are provided in Figures 7c,d. Since the two‐centre ICOBI directly corresponds to the bond order, the ICOBI^(2)^ values of 1.11 for the two types of Se–Se bonds in the Se_4_‐unit show that these bonds can be understood as covalent single bonds. The four‐centre ICOBI^(4)^ value given in the right part of the Figure shows that there is also a significant four‐centre interaction, some delocalisation across the ring. The highest orbital‐wise contribution to this interaction of a $4p_{z}\frac{\;4\mathrm{pz}\;4\mathrm{pz}}{}4p_{z}$ character. Here, the 4p_z_ orbitals are arranged perpendicular to the bonds in the Se_4_ unit.

LOBSTER also allows for the calculation of molecular (instead of atomic) orbitals of a defined molecular unit [68]. The MO‐DOS as well as the MO‐scheme with the corresponding molecular orbitals for the Se_4_ unit are given in Figure 8b. An even more detailed MO‐scheme is presented in Figure 8d to show the atomic‐orbital contributions to the MOs. The molecular orbital formation energy (MOFE) in Figure 8c can be understood as a COHP analogue that is partitioning of the Kohn‐Sham energy but molecular‐orbital like. Hence, MOFE measures the energetic contribution of each atomic orbital to a molecular orbital, plotted here such that bonding MOs appear to the right of Figure 8c and antibonding MOs to the left. The integrated MOFE values (IMOFE) corresponding to the diverse interactions forming the molecular orbitals are also provided in Figure 8c.

**FIGURE 8:**
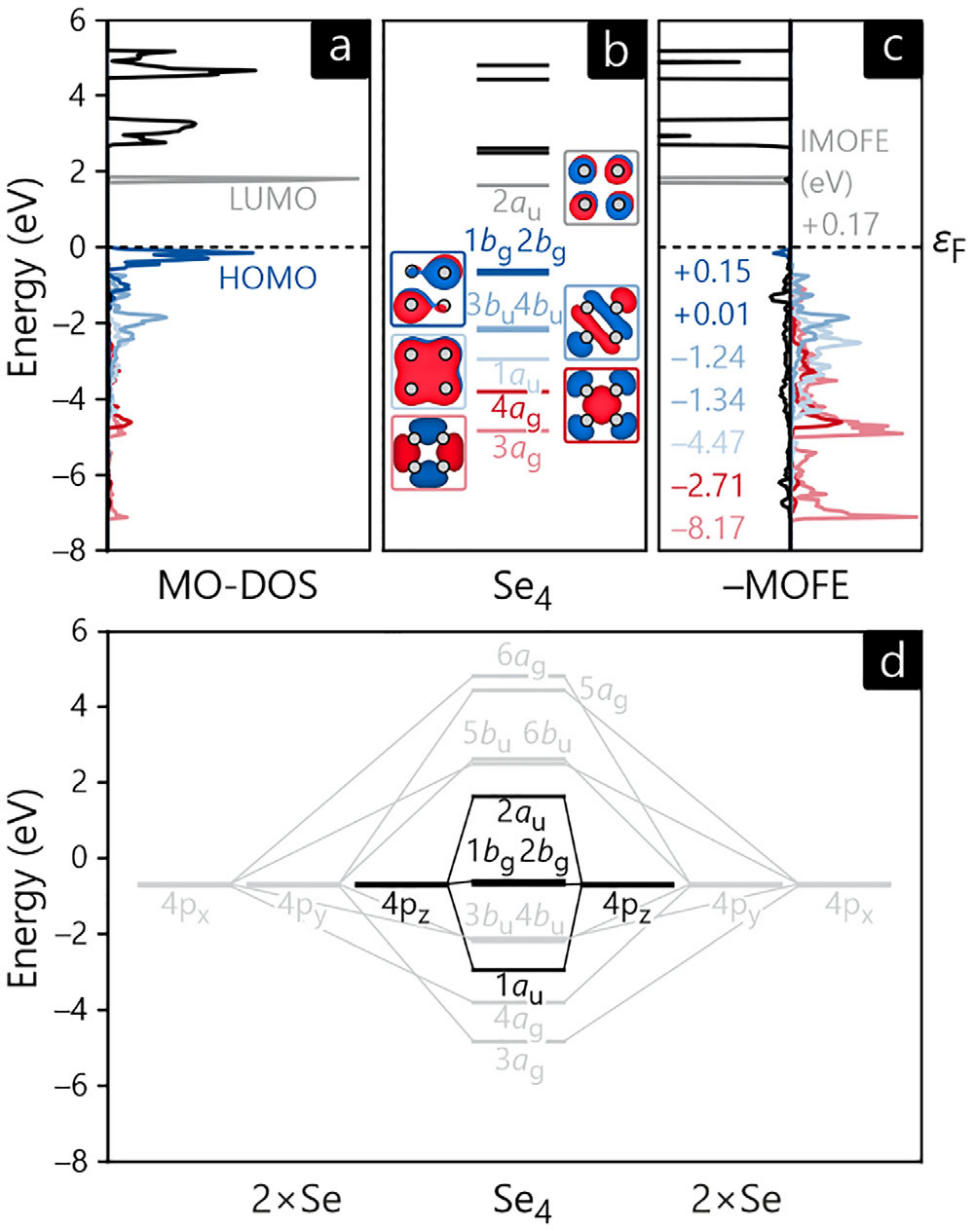
(a) Molecular Orbital‐resolved DOS, (b) Molecular Orbital (MO) scheme with visualized molecular orbitals, (c) MOFE diagram with IMOFE values for the Se_4_‐unit and (d) MO scheme with AO contributions.

A more detailed analysis of the MOFE diagram in Figure 8c reveals that the 1*b*_g_ and 2*b*_g_ levels between −2 eV and the valence‐band maximum appear on the left side of the plot indicating a slightly antibonding/nonbonding character. The corresponding IMOFE values of +0.15 eV and +0.01 eV reveal a minor destabilization caused by the formation of these molecular orbitals. These levels can be assigned to the selenium “lone pairs”, which show up as the HOMO levels of the Se_4_ unit. The more detailed MO scheme in Figure 8d yields that the 1*b*_g_ and 2*b*_g_ are formed by the 4p_z_ orbitals and have a *π*‐character. The 4p_z_ orbitals also form two additional *π*‐character MOs. These are the 1*a*_u_ as well as the antibonding 2*a*_u_. The Löwdin gross population of the Se_4_‐unit (Table 2) shows that this unit follows the *Aufbau principle* as indicated by the doubly occupied valence levels below the Fermi level and (almost) empty conduction levels.

**TABLE 2:** MO–energy, IMOFE, and Löwdin gross orbital population (GOP) for the Se_4_‐unit

Molecular Orbital	MO energy (eV)	IMOFE (eV)	Löwdin GOP
2*a*_u_	+1.63	+0.17	0.07
2*b*_g_	−0.60	+0.15	1.99
1*b*_g_	−0.69	+0.01	1.99
4*b*_u_	−2.12	−1.24	1.99
3*b*_u_	−2.19	−1.34	1.99
1*a*_u_	−2.94	−4.47	1.98
4*a*_g_	−3.80	−2.71	1.97
3*a*_g_	−4.83	−8.17	1.99

### 2.8 Magnetic Susceptibility Studies

The temperature‐dependent magnetic susceptibilities of Se_4_[B(S_2_O_7_)_2_]_2_(SO_3_) and Se_4_[S_4_O_13_] are shown in Figure 9. Both compounds are diamagnetic over the whole temperature range with room temperature values of −6(1) × 10^−4^ emu mol^−1^ for Se_4_[B(S_2_O_7_)_2_]_2_(SO_3_) and −6.0(2) × 10^−4^ emu mol^−1^ for Se_4_[S_4_O_13_]. The susceptibility increase at low temperatures can be attributed to tiny amounts of paramagnetic impurities (so‐called Curie tails). The Se_4_[B(S_2_O_7_)_2_]_2_(SO_3_) sample seems to have an additional diamagnetic impurity that shows up below 50 K, most likely arising from the reaction with the capsule (*vide ultra*).

**FIGURE 9:**
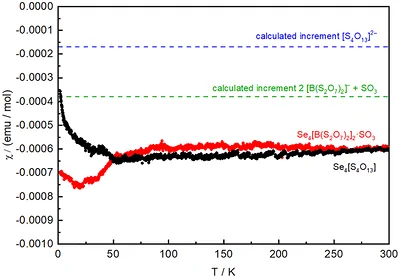
Temperature dependence of the magnetic susceptibi‐lities of Se_4_[B(S_2_O_7_)_2_]_2_(SO_3_) and Se_4_[S_4_O_13_] measured with an applied external magnetic field of 10 kOe. For comparison, the summed diamagnetic increments [69] for [S_4_O_13_]^2–^ and 2[B(S_2_O_7_)_2_]^–^ + SO_3_ are shown by dotted lines.

To classify the room temperature values of Se_4_[B(S_2_O_7_)_2_]_2_(SO_3_) and Se_4_[S_4_O_13_], we compare the experimental data with the sums of diamagnetic increments [69]. Since no increment is available for the Se_4_^2+^ cation, we calculated the sums for the anions [S_4_O_13_]^2–^ and 2[B(S_2_O_7_)_2_]^–^ + SO_3_. These values are drawn as dotted lines in Figure 9. They are of the same order of magnitude as the experimental ones. The diamagnetic contributions of the Se_4_^2+^ cations then push the susceptibilities to lower values, hence in good agreement between the experimental values and the increments.

### 2.9 Optical Spectroscopy

To quantify the yellow color of the compounds, we recorded powder reflectance spectra of Se_4_[B(S_2_O_7_)_2_]_2_, Se_4_[B_2_(SO_4_)_2_(S_2_O_7_)_2_]‐I, Se_4_[B_2_(SO_4_)_2_(S_2_O_7_)_2_]‐II, and Se_4_[B_2_(SO_4_)_4_] from 200 to 750 nm (Figure 10). The spectra are dominated by three very intense broad bands with one ranging up to 500 nm into the visible regime; this is consistent with the yellow color of the powders based on a strong absorption in the blue to violet regime. These broad bands originate from π*←π transitions within Se_4_^2+^ and agree well with results of the DFT calculations for Se_4_[B_2_(SO_4_)_4_] considering the systematic underestimation of the band gap by this method (up to 40%). The first absorption band peaking around 450 nm can be assigned to a 2*a*_u_←2*b*_g_/1*b*_g_ transition while the transition around 330 nm can be assigned to the 5*b*_u_/6*b*_u_←2*b*_g_/1*b*_g_ transition. Spectroscopic data on Se_4_^2+^ cations in solid samples are scarce, as listed in Table 3 [18, 60]. With absorption maxima located between 440 to 460 nm the coordination strength of borosulfates resembles that of hexafluoridoarsenates. A comparison between the spectra shows a small red shift from Se_4_[B(S_2_O_7_)_2_]_2_ to Se_4_[B_2_(SO_4_)_4_]. These results are in accordance with earlier findings in borosulfates where compounds with terminal disulfate groups compared to solely sulfate groups show a slightly weaker coordination strength. The additional broad band around 700 nm presumably originates from transitions within Se_8_^2+^ ions occasionally formed from unreacted Se_4_^2+^ ions yielding a greenish hue – which happens if the sample gets into contact with reducing agents [18]. Essentially, this suggests that we might see a borosulfate featuring Se_8_^2+^ soon.

**FIGURE 10:**
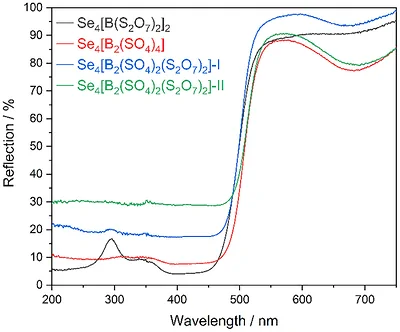
Powder reflectance spectra of Se_4_[B(S_2_O_7_)_2_]_2_ (black), Se_4_[B_2_(SO_4_)_4_ (red), Se_4_[B_2_(S_2_O_7_)_2_(SO_4_)_2_]‐I (blue) and Se_4_[B_2_(S_2_O_7_)_2_(SO_4_)_2_]‐II (green) recorded between 200 and 750 nm.

**TABLE 3:** Diffuse reflectance spectra and electronic absorption of Se_4_^2+^ in various compounds reported previously compared to Se_4_[B(S_2_O_7_)_2_]_2_

Species	Color	π→π* transition 2*b*_g_/1*b*_g_ → 2*a_u_*	π→π* transition 2*b*_g_/1*b*_g_ → 5*b*_u_/6*b*_u_	References
Se_4_^2+^ (solution)	yellow	410	320	[20]
Se_4_[Sb_2_F_4_][Sb_2_F_5_][SbF_6_]_5_	yellow	413	382	[60]
Se_4_[AsF_6_]_2_	yellow	440	385	[60]
Se_4_[B(S_2_O_7_)_2_]_2_	yellow	444	392	this work
Se_4_[AlCl_4_]_2_	orange	488	430	[60]
Se_4_[HS_2_O_7_]_2_	orange	524	428	[60]

### 2.10 Infrared Spectroscopy

In Figure 11, the infrared spectra of Se_4_[B(S_2_O_7_)_2_]_2_, Se_4_[B_2_(SO_4_)_2_(S_2_O_7_)_2_]‐I, Se_4_[B_2_(SO_4_)_2_(S_2_O_7_)_2_]‐II, and Se_4_[B_2_(SO_4_)_4_] between 1500 and 400 cm^−1^ are displayed — full spectra are shown in Figure S4 — and reveal bands typical for borate and sulfate tetrahedra. The assignments in the recorded spectra were achieved by comparing the bands to experimental and calculated data of Ba[B(S_2_O_7_)_2_], a borosulfate that also features S–O–S bridges and even contains the same [B(S_2_O_7_)_2_]^2–^ anion [48]. Above 1500 cm^−1^, two broad bands centred around 2930 and 2425 cm^−1^ occur and can be assigned to small amounts of adhesive sulfuric acid [70]. The bands between 1430 and 1330 cm^−1^ can be assigned to asymmetric stretching vibrations *ν*_as_(S–O). Symmetric stretching vibrations *ν*_s_(S–O_t_) of terminal oxygen atoms are found between 1215 and 1185 cm^−1^. In the region from 1105 to 940 cm^−1^, symmetric and asymmetric stretching vibrations *ν*_s/as_(B–O) in (BO_4_) tetrahedra occur. Bending vibrations *δ*(O–B–O) are found in the region 920–870 cm^−1^. The bands between 770 and 640 cm^−1^ can be assigned to stretching vibrations *ν*_s_(S–O_b_) of bridging oxygen atoms within the (S_2_O_7_) groups, confirming the presence of S–O–S bridges. Lastly, between 615 and 460 cm^−1^, bending vibrations of the (BO_4_) *δ*(O–B–O) and (SO_4_) tetrahedra *δ*(O–S–O) can be found. Due to the limited range of the spectrometer, Se–Se vibrations could not be further evaluated. These bands are typically found around 320 cm^−1^ [60].

**FIGURE 11:**
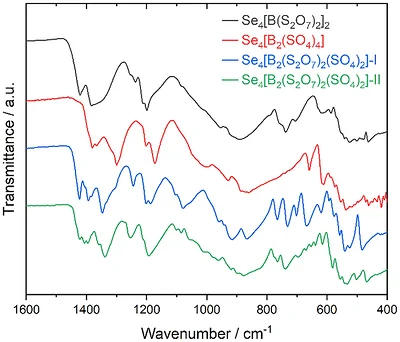
Infrared spectra of Se_4_[B(S_2_O_7_)_2_]_2_ (black), Se_4_[B_2_(SO_4_)_4_] (red), Se_4_[B_2_(SO_4_)_2_(S_2_O_7_)_2_]‐I (blue), and Se_4_[B_2_(SO_4_)_2_(S_2_O_7_)_2_]‐II (green) between 1600 and 400 cm^−1^.

### 2.11 Thermal Analysis

The transformation of borosulfates into other borosulfates is well known. Compounds comprising S–O–S bridges typically decompose towards compounds containing B–O–S bridges and finally B–O–B bridges as observed in the series of the barium borosulfates Ba[B(S_2_O_7_)_2_]_2_, Ba[B_2_(SO_4_)_4_], and Ba[B_2_O(SO_4_)_3_] [48]. However, it is important to note that these transformations do not occur in all cases, but they can depend on the environment during the decomposition process; for instance, under nitrogen atmosphere, the decomposition of Sr[B_2_(SO_4_)_4_] immediately leads to the formation of SrSO_4_ while in mother liquor the formation of Sr[B_2_O(SO_4_)_3_] is observed [53]. Similarly, during the decomposition of Sr[B_2_(SO_4_)_3_(S_2_O_7_)] in a capillary, monitored by X‐ray diffraction analysis, Sr[B_2_O(SO_4_)_3_] was verified as an intermediate [71]. Hence, high sulfate or SO_3_ concentrations in a closed environment during decomposition foster the formation of further borosulfates.

A powerful tool to identify the composition of crystalline products with increasing temperature is the temperature‐programmed powder X‐ray diffraction (TPPXRD). These experiments were carried out in a capillary and revealed a structural relationship between all five borosulfates of this work. The diffraction patterns in Figure 12a indicate a thermal stability of Se_4_[B(S_2_O_7_)_2_]_2_ until 80°C. At 100°C, with the release of two moles SO_3_ the *bis*‐disulfatoborat groups condense to form larger zero‐dimensional anions, still featuring S–O–S bridges:

(1) $${\text{Se}}_{4}{\left[B{\left(S_{2}O_{7}\right)}_{2}\right]}_{2}\to {\text{Se}}_{4}\left[B_{2}{\left({\text{SO}}_{4}\right)}_{2}{\left(S_{2}O_{7}\right)}_{2}\right]-I+2{\text{SO}}_{3(g)}$$

**FIGURE 12:**
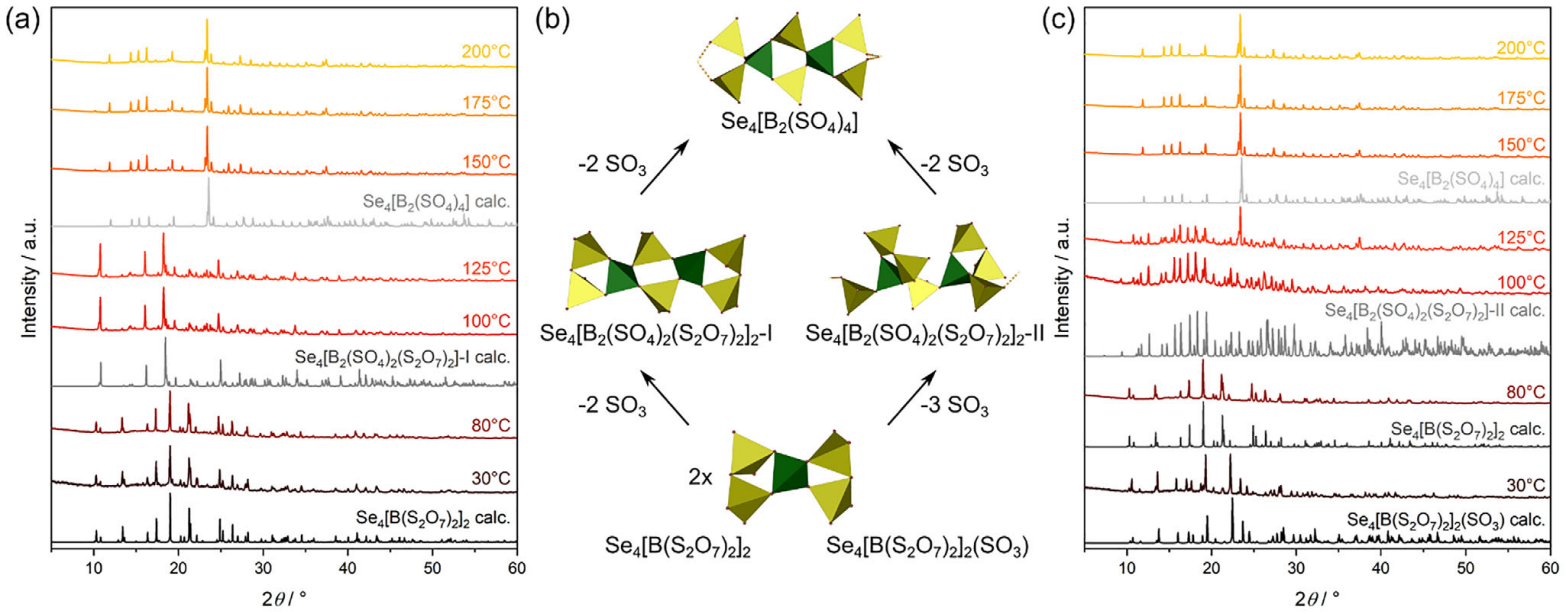
TPPXRD patterns of (a) Se_4_[B(S_2_O_7_)_2_]_2_ and c) Se_4_[B(S_2_O_7_)_2_]_2_(SO_3_). (b) Schematic representation of the stepwise condensation from the [B(S_2_O_7_)_2_]^−^ anion in Se_4_[B(S_2_O_7_)_2_]_2_ and Se_4_[B(S_2_O_7_)_2_]_2_(SO_3_) dependent on the SO_3_ content within the capillary over the zero‐ and one‐dimensional anions [B_2_(SO_4_)_2_(S_2_O_7_)_2_]^−^ of the two polymorphs in Se_4_[B_2_(SO_4_)_2_(S_2_O_7_)_2_] to the final decomposition product Se_4_[B_2_(SO_4_)_4_] with the [B_2_(SO_4_)_4_]^−^ anion by the release of further two moles of SO_3._

Subsequently, Se_4_[B_2_(SO_4_)_2_(S_2_O_7_)_2_]‐I decomposes at 150°C to form Se_4_[B_2_(SO_4_)_4_], a one‐dimensional borosulfate comprising B–O–S bridges. Hence, this compound is stable up to 200°C:

(2) $${\text{Se}}_{4}\left[B_{2}{\left({\text{SO}}_{4}\right)}_{2}{\left(S_{2}O_{7}\right)}_{2}\right]-I\to {\text{Se}}_{4}\left[B_{2}{\left({\text{SO}}_{4}\right)}_{4}\right]+2{\text{SO}}_{3(g)}$$

In comparison, Figure 13c shows the decomposition process starting from the related selenium *bis*‐borodisulfate Se_4_[B(S_2_O_7_)_2_]_2_(SO_3_) still containing molecular SO_3_ units. In a first step, at 80°C the incorporated molecular SO_3_ is evaporated to transform into the SO_3_‐free analogue:

(3) $${\text{Se}}_{4}{\left[B{\left(S_{2}O_{7}\right)}_{2}\right]}_{2}\left({\text{SO}}_{3}\right)\to {\text{Se}}_{4}{\left[B{\left(S_{2}O_{7}\right)}_{2}\right]}_{2}+{\text{SO}}_{3(g)}$$

**FIGURE 13:**
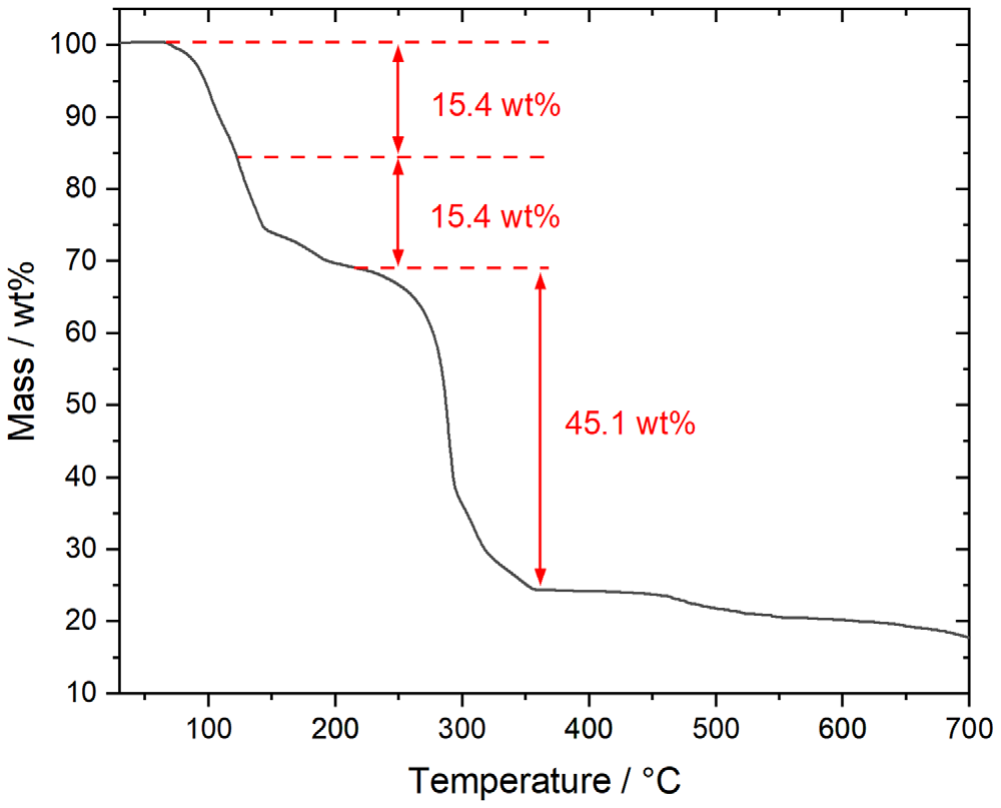
Thermal analysis of Se_4_[B(S_2_O_7_)_2_]_2_ in a nitrogen atmosphere with a heating rate of 5 K·min^−1^.

Like in the earlier discussed case, Se_4_[B(S_2_O_7_)_2_]_2_ decomposition starts at 100°C, however, now to form polymorph II of Se_4_[B_2_(SO_4_)_2_(S_2_O_7_)_2_]. This time, the [B(S_2_O_7_)_2_]^2−^ units condense to form chains:

(4) $${\text{Se}}_{4}{\left[B{\left(S_{2}O_{7}\right)}_{2}\right]}_{2}\to {\text{Se}}_{4}\left[B_{2}{\left({\text{SO}}_{4}\right)}_{2}{\left(S_{2}O_{7}\right)}_{2}\right]\text{-II}+2{\text{SO}}_{3(g)}$$

Se_4_[B_2_(SO_4_)_2_(S_2_O_7_)_2_]‐II starts to decompose around 125°C. Minor reflections can be assigned to Se_4_[B_2_(SO_4_)_4_] and indicate a lower thermal stability compared to the first polymorph which is in accordance with the general trend observed within borosulfates where the thermal stability decreases with increasing dimensionality [72]:

(5) $${\text{Se}}_{4}\left[B_{2}{\left({\text{SO}}_{4}\right)}_{2}{\left(S_{2}O_{7}\right)}_{2}\right]\text{-II}\to {\text{Se}}_{4}\left[B_{2}{\left({\text{SO}}_{4}\right)}_{4}\right]+2{\text{SO}}_{3(g)}$$

Finally, Se_4_[B_2_(SO_4_)_4_] decomposes above 200°C to form an amorphous residue consistent with the first TPPXRD experiment. It seems that different SO_3_ partial pressures in the closed system allow for two alternative decomposition processes. Both differ only regarding an additional SO_3_ unit per sum formula of the starting material. This transformation from one compound to another, where both contain S–O–S bridges, is a unique feature of this system which was observed for the first time in borosulfate chemistry.

To verify the results of the TPPXRD experiments, we further employed thermogravimetric analyses (TGA) (Figure 13). Due to the volatility of SO_3_ in Se_4_[B(S_2_O_7_)_2_]_2_(SO_3_), already evaporating at room temperature over time, a conclusive measurement was not possible. Considering the SO_3_‐free compound, the mass losses from Equations (1) and (2) of 14.9 % and 15.6 % agree well with the evaporation of two moles SO_3_ per step (Δ*m*_calc_ = 15.4%) and the formation of Se_4_[B_2_(SO_4_)_4_]. The intermediate formation of one of the polymorphs overlays with its decomposition caused by the small stability regime leading to no proper development of a plateau. The residue after the decomposition of Se_4_[B_2_(SO_4_)_4_] was not unambiguously determined as the powder was amorphous. Nevertheless, the red body color hints to the formation of red selenium which is typical to be formed at these temperatures; this agrees with the PXRD results (Equation (5)).

## 3 Conclusion

The stabilization of polycations of group 16 elements in the solid state almost exclusively occurs in Lewis acidic (transition) metal (oxo)halides that provide weakly coordinating anions, a precondition for their stabilization. In this contribution, we report on six compounds stabilising the Se_4_^2+^ cation in a Brønsted acidic, purely oxoanionic coordination environment representing a significant contribution towards the stabilization of these compounds since Se_4_[HS_2_O_7_]_2_ was synthesized in 1968.

Herein, we added the first five borosulfates hosting Se_4_^2+^ ions. Both Se_4_[B(S_2_O_7_)_2_]_2_(SO_3_) and Se_4_[B(S_2_O_7_)_2_]_2_ feature the well‐known [B(S_2_O_7_)_2_]^–^ anion; the former can be converted into the latter by evaporation of the volatile SO_3_. Se_4_[B_2_(SO_4_)_2_(S_2_O_7_)_2_] forms two polymorphs with rare zero‐ and one‐dimensional anions. Finally, we synthesised Se_4_[B_2_(SO_4_)_4_], a borosulfate comprising well‐known SiS_2_‐like chains, substituting B by Si and (SO_4_) by S. In this study, we also solved the crystal structure of Se_4_[S_4_O_13_], a compound discovered in 1968 but not structurally characterized to date. The compound exhibits the uncommon tetrasulfate anion.

It is well known that the color of compounds containing Se_4_^2+^ cations red shifts from yellow to red with increasing coordination strength of the anion. Accordingly, the yellow color observed for all five borosulfates and the tetrasulfate confirms the weak coordination of borosulfates and polysulfates. Therefore, we recorded the very first powder reflectance spectra of solids containing Se_4_^2+^ and observed, among others, a strong absorption in the blue regime responsible for the compound's color. Based on LOBSTER calculations this band can be attributed to π*←π transitions within the selenium ring. This transition energy depends on the coordination strength of the chemical surrounding and confirms the relation between color and coordination strength. LOBSTER also provided insights into the bonding situation and charge distribution in Se_4_^2+^. These results are in excellent accordance with magnetic measurements on Se_4_[B(S_2_O_7_)_2_]_2_(SO_3_) and Se_4_[S_4_O_13_], confirming their diamagnetism, hence, the absence of unpaired electrons in the selenium cation.

Moreover, TPPXRD measurements reveal that the borosulfates of this study form sequentially with increasing decomposition temperature, enabling their selective synthesis. For the first time in borosulfates, the loss of an SO_3_ entity could be directly tracked. The borosulfates Se_4_[B(S_2_O_7_)_2_]_2_(SO_3_) and Se_4_[B(S_2_O_7_)_2_]_2_, differing only by the presence of one SO_3_ molecule decompose to the common intermediate phase, Se_4_[B_2_(SO_4_)_2_(S_2_O_7_)_2_], which was observed in two distinct modifications. These modifications can be selectively obtained depending on whether the SO_3_‐containing or SO_3_‐free precursor is thermally decomposed. The further decomposition yields the same phase, that is Se_4_[B_2_(SO_4_)_4_]. This colorful and diverse chemistry suggests that there is a lot more to come regarding compounds containing group 16 oligocations.

## Author Contributions

**Erich Turgunbajew**: conceptualization, methodology, data curation, writing – review and editing, writing – original draft, visualization, validation, investigation, project administration. **Marios Symeonidis**: investigation, visualization, writing – review and editing. **Jörn Bruns**: writing – review and editing, visualization, validation, investigation, project administration, supervision, resources, writing – original draft. **Sara N. Özbay**: writing – review and editing, investigation. **Jan Langwald**: investigation, visualization, writing – review and editing. **Mathias S. Wickleder**: writing – review and editing, visualization, validation, resources, writing – original draft. **Robert E. Dinnebier**: investigation, writing – review and editing, visualization, resources. **Linda S. Reitz**: visualization, writing – review and editing, investigation. **Richard Dronskowski**: investigation, writing – review and editing, software, visualization, validation, resources, writing – original draft. **Lars Schumacher**: visualization, investigation, writing – review and editing. **Rainer Pöttgen**: writing – review and editing, data curation, funding acquisition, visualization, validation, resources, writing – original draft. **Henning A. Höppe**: conceptualization, validation, funding acquisition, project administration, writing – original draft, writing – review and editing, supervision, data curation, formal analysis, resources.

## Conflicts of Interest

The authors declare no conflicts of interest.

## Supporting information



A detailed description of the experimental as well as computational methodology is provided in the Supporting information. The authors have cited additional references within the Supporting Information [66, 67, 73, 74, 75, 76, 77, 78, 79, 80, 81, 82, 83, 84, 85, 86, 87, 88, 89, 90, 91, 92, 93, 94, 95, 96, 97, 98]. **Supporting File**: anie74838‐sup‐0001‐SuppMat.pdf.

## Data Availability

The data that support the findings of this study are available from the corresponding author upon reasonable request.
