# Supplementary material for: Borosulfates Hosting Se4 2+ Ions—Crystal Structures, Chemical Bonding, Optical Spectroscopy, and Magnetism
Source: Angew Chem Int Ed Engl. 2026 Sep 19;65(45):e4300417. doi: 10.1002/anie.4300417 (PMC13725582; doi:10.1002/anie.4300417)
Supplement: Supplementary file 1 [file ANIE-65-e4300417-s001.pdf]

Angewandte Chemie International Edition

Supporting Information

Borosulfates Hosting Se_4^{2+} Ions – Crystal Structures, Chemical Bonding, Optical Spectroscopy and Magnetism

Erich Turgunbajew, Marios Symeonidis, Jörn Bruns, Sara N. Özbay, Jan Langwald, Mathias S. Wickleder, Robert E. Dinnerbier, Linda S. Reitz, Richard Dronskowski, Lars Schumacher, Rainer Pöttgen, Henning. A. Höppe

Experimental Section

Synthesis

$Se_4[B_2(SO_4)_2(S_2O_7)_2]$-I and $Se_4[B_2(SO_4)_2(S_2O_7)_2]$-II: Se (0.438 g, 5.5 mmol) and B_2O_3 (0.096 g, 1.4 mmol, Sigma Aldrich) were transferred into a silica ampoule (length: 12 cm, diameter: 1 cm, wall thickness: 0.1 cm). After the addition of 1 mL oleum (65% SO_3, Merck) the ampoules were torch sealed. Prior to the thermal treatment at the respective reaction temperatures, in both cases an aging step of the mother liquor was necessary. Therefore, the ampoules were stored in a drying chamber for one day, with the temperature set to 50°C. After letting the ampoules cool to room temperature, they were again placed in a drying chamber at the respective reaction temperature. Crystals of $Se_4[B_2(SO_4)_2(S_2O_7)_2]$-I were obtained when the ampoules were stored for several days at 50°C, while $Se_4[B_2(SO_4)_2(S_2O_7)_2]$-II was formed when the ampoules were stored at 110°C. Following the decantation of the excess oleum, the ampoules were frozen with liquid nitrogen and eventually opened. (**Caution!** During and even after the reaction, the ampoules are under remarkable pressure and must therefore be handled with great care, e.g., they must be cooled with liquid nitrogen prior to opening.) The moisture sensitive products were initially washed with hot oleum to remove excess selenium. Subsequently, to remove the sulfuric acid the product was washed several times with thionyl chloride (99.7%, Acros Organics) using a setup under nitrogen atmosphere. For further investigation the yellow greenish products were transferred into an argon filled glovebox.

$Se_4[B_2(SO_4)_4]$: Yellow greenish crystals of $Se_4[B_2(SO_4)_4]$ were formed via the thermal decomposition of either $Se_4[B_2(SO_4)_2(S_2O_7)_2]$-I and $Se_4[B_2(SO_4)_2(S_2O_7)_2]$-II. Following the synthesis of either compound, the closed ampoules were placed in a muffle furnace, and the following temperature program was applied: heating to 200°C with 50°K/h, holding the temperature for 12 h, and cooling to room temperature.

$Se_4[B(S_2O_7)_2]_2$: $Se_4[B(S_2O_7)_2]_2(SO_3)$ is stored for several weeks in the glovebox.

***Caution*:** SO_3 is a very strong oxidizer and should be handled carefully. During and even after the reaction, the ampoule might be under remarkable pressure. It is mandatory to cool the ampoule with liquid nitrogen prior to opening.

$Se_4[B(S_2O_7)_2]_2(SO_3)$: Se (50.37 mg; 0.6378 mmol, Acros Organics) and B_2O_3 (100.37 mg; 1.4417 mmol, BLDpharm) were loaded into a thick-walled glass ampoule (length = 200 mm, ø = 16 mm, wall thickness = 1.8 mm) and subsequently attached to an apparatus for the generation, handling and titration of SO_3 under strict exclusion of oxygen and moisture. Oleum (65 % SO_3, Merck) was added dropwise to an excessive amount of P_4O_{10} (97 %, Merck). The mixture was heated with an aluminum block to 403 K to transfer the freshly generated SO_3 into the gaseous phase. During the whole process the pressure must be continuously monitored by a Teflon-lined manometer, attached above the dropping funnel. The gaseous SO_3 condenses and accumulates in a burette, as a part of the specifically designed apparatus. For the synthesis of the title compound 0.6 mL freshly distilled SO_3 was added to the attached ampoule after opening a Teflon-lined valve. The ampoule was torch sealed at a height of approx. 200 mm under vacuum (0.01 mbar). The ampoule was placed in a cylindrical resistance-furnace with a variable temperature program. The ampoule was heated to 393 K within 24 h, then the temperature was held for 48 h and subsequently cooled to 298 K within 90 h. The product crystallized as yellow, diamond-shaped crystals at the inner walls of the ampoule.

$Se_4[S_4O_{13}]$: 95.0 mg (1.20 mmol, 1.22 eq.) selenium (99+%, Thermo Fischer Scientific) and 156 mg (0.98 mmol, 1.00 eq.) bromine (99+%, Thermo Fischer Scientific) were filled into a preheated glass ampoule (3.3 borosilicate). 0.82 mL SO_3 (20.48 mmol, 20.90 eq.) were condensed on top, and the ampoule was sealed under reduced pressure ($1 \cdot 10^{-3}$ mbar) with a gas burner. The reaction vessel was heated to 80 °C within 24 h, dwelled at this temperature for 48 h and cooled down to room temperature within 90 h. After cooling, bright yellow-greenish platelets grew.

Single crystal X-ray diffraction

Immediately after opening the ampoules, single crystals of $Se_4[B_2(SO_4)_4]$, $Se_4[B_2(SO_4)_2(S_2O_7)_2]$–I and $Se_4[B_2(SO_4)_2(S_2O_7)_2]$–II were transferred from the mother liquor into perfluorinated polyether (MiTeGen, LV CryoOil). Suitable crystals were picked under a polarization microscope, mounted onto a MicroMount (MiTeGen) and directly transferred into a cold nitrogen gas stream (Oxford Cryostream Plus). Single crystal X-ray diffraction data was collected on a Bruker D8 Venture diffractometer with MoK_α radiation (0.71073 Å) equipped with a PHOTON-100 detector. Absorption correction was performed via the multi-scan method.[1] The crystal structures were solved by Direct Methods using the SHELXS program[2] and refined by full-matrix least-squares technique with the SHELXTL program.[3]

The single crystal structure determination for $Se_4[S_4O_{13}]$ was performed on a Bruker D8 VENTURE KAPPA diffractometer with a microfocus sealed tube using a multilayer mirror as the monochromator and a Bruker PHOTON III detector. As characteristic X-ray radiation MoK_α (λ = 71.073 pm) was used for the measurements. Before the measurements, the crystals were prepared in perfluorinated ether (Fomblin® YR-180) and selected with the aid of a light microscope with a polarization filter. The crystals were fixed on a micromount with a 150 µm polymer loop and adjusted to the X-ray beam with optional cooling to 100 K. The images with the intensity data were processed using the software APEX5.[4] The frames were integrated with the Bruker SAINT software package using a narrow frame algorithm. Absorption effects were

corrected using SADABS for the multi-scan absorption correction.[5,6] The structure solution and refinement were done with the software OLEX2.[7] Dual methods using SHELXT were used for structure solution and full-matrix least-squares methods against F2 using SHELXL for the refinement.[3,8] The structure was refined as an inversion twin using a twin law with a batch scale factor (BASF) of 0.425(7).

Before opening, the SO_3-containg side of the ampoule was cooled with liquid nitrogen. After opening the ampoule, several crystals of $Se_4[B(S_2O_7)_2]_2(SO_3)$ were transferred immediately into perfluoroalkylether oil (Fomblin® YR-180, ABCR chemicals, Karlsruhe, Germany). A suitable yellow, diamond-shaped crystal was isolated and mounted onto a polymer loop (MicroMounts, MiTeGen LLC, New York, USA) under a polarization microscope and quickly placed into a cold N_2 stream (111 K) inside the diffractometer (Bruker D8 Venture κ, Bruker AXS GmbH, Karlsruhe, Germany) equipped with a Bruker PHOTON III detector and a microfocus sealed tube using a multilayer mirror as a monochromator. As X-ray source Mo-K_α radiation (71.073 pm) was used. After unit cell determination the reflection intensities were collected using APEX5.[4] The integration was performed using SAINT and a multi-scan absorption correction was applied using SADABS.[6] The structure solution was done in the software Olex2 using intrinsic phasing (SHELXT).[3,7,8] The structural model was refined by least squares methods using SHELXL. The structure was solved in the monoclinic crystal system in space group *C*2/*c* (no. 15). All atoms were refined with anisotropic displacement parameters.

Powder X-ray diffraction and Rietveld refinement

The samples were ground and filled into Hilgenberg glass capillaries (outer diameter 0.3 mm, wall thickness 0.01 mm). The PXRD patterns were recorded with a D8 Venture in transmission geometry using Cu-K_α radiation (1.54184 Å), a 1D LynxEye detector and a nickel filter to suppress K_β radiation. Temperature-programmed PXRD measurements were performed on the same device using an oven attachment. Further powder X-ray diffraction and Rietveld refinement P-XRD data was collected on a Stoe Stadi-P powder diffractometer with a Mythen detector in Debye−Scherrer geometry by using Mo-$K_{\alpha 1}$ radiation of λ = 0.70930 Å. The Rietveld refinement was performed using the software TOPAS-Academic (64 V6).[9] The refinement process included the scale factor and the lattice parameters, along with corrections for zero shift, specimen displacement, and background. The isotropic atomic displacement parameters were set to 1 for all atoms. Peak profiles were described using the pseudo-Voigt function of Thompson, Cox, and Hastings,[10] with the profile shape parameters *U*, *V*, *W*, and y refined accordingly.

Structure determination and Rietveld refinement of $Se_4[B(S_2O_7)_2]_2$

For indexing of the powder pattern of $Se_4[B(S_2O_7)_2]_2$ at T = 298 K, the program TOPAS version 7[9] was used. Indexing was performed by iterative use of singular value decomposition (LSI),[11] leading to a *C*-centred monoclinic unit cell with unit cell parameters of a = 19.1728(2) Å, b = 10.1834(1) Å, c = 14.5287(2) Å, and β = 116.521(1)° (V = 2538.15(5) Å^3). The possible space group could be determined as *Cc or C*2/*c* from the observed extinction rules from which the latter was confirmed after structure determination. The number of formula units per unit cell could be estimated to Z = 4 from volume increments or one $B(S_2O_7)_2$ and on two Se atoms in the asymmetric unit.

The peak profiles and precise lattice parameters of the powder patterns of DBT were first determined by a Pawley fit[12] using the fundamental parameter (FP) approach of TOPAS.[13] For the modelling of the background, Chebychev polynomials of fifth order and a broad Lorentzian peak to account for the amorphous hump of the glass capillary were employed. Several very small reflections of an unknown impurity phase were modelled by single peak fitting. The refinement converged quickly.

Structure determination of $Se_4[B(S_2O_7)_2]_2$ was performed in an iterative manner by the global optimization method of simulated annealing in real space using the TOPAS program.[14,15] Two rigid bodies in *Z*-matrix notation, describing a $B(S_2O_7)_2$ molecule and a quadratic Se_4 unit (half occupancy) were defined using the bond lengths, bond angles and torsion angle results from the previously solved crystal structure of $Se_4[B(S_2O_7)_2]_2(SO_3)$. The two rigid bodies were subjected to the simulated annealing process with 12 external degrees of freedom, 2×3 translational and 2×3 rotational. Quickly a reasonable match with the powder pattern was achieved. Unsurprisingly the centre of mass of the Se_4 unit is located on a centre of symmetry.

For the Rietveld refinement,[16] all individual atoms were refined. Two selenium atoms and 19 atoms of the $B(S_2O_7)_2$ unit, all on general positions. The $B(S_2O_7)_2$ unit was further stabilized by slack soft constraints (restraints) for the 20 bond lengths and the 30 bond angles within the tetrahedra to avoid meaningless positional changes. The final Rietveld refinement (using the TOPAS program) is shown in Fig. S9. Agreement factors are listed in Tab. S2. The atomic coordinates are given in Tab. S3.

UV-Vis spectroscopy

The diffuse reflectance spectra were recorded at room temperature using a Cary 300 Scan UV-Vis spectrometer (Varian) equipped with a D1 gas-discharge (200 nm-350 nm), a tungsten-halogen light source (350 nm-800 nm) and an Ulbricht sphere detector (increment 1 nm, scan rate: 120 nm · min^{-1}).

Infrared spectroscopy

The infrared spectra of $Se_4[B_2(SO_4)_4]$ were recorded using an EQUINOX 55 FT-IR spectrometer (Bruker) equipped with a platinum ATR unit in the range of 4000-400 cm^{-1} and a resolution of 4 cm^{-1} with 32 scans per measurement.

Physical property measurements

Powdered samples of $Se_4[B(S_2O_7)_2]_2(SO_3)$ and $Se_4[S_4O_{13}]$ were filled in polypropylene capsules. The samples were placed in a brass sample holder and attached to the sample holder of a Dynacool physical property measurement system (PPMS) by Quantum Design using the vibrating sample magnetometer (VSM) option. They were investigated in a temperature range of 2-300 K at external fields of 10 kOe. $Se_4[B(S_2O_7)_2]_2(SO_3)$ has shown some reaction with the capsule, resulting in blackening of the material. Plotting the data was done with OriginPro 2024[17] and the graphical editing with the program CorelDraw 2024.[18]

Computational Details

Structural optimizations were carried out using the Vienna ab initio Simulation Package (VASP, v.5.4).[19–22] The simulations were considered converged for energetic differences of 10^{-8} eV for electronic and 10^{-6} eV·Å^{-1} for ionic optimizations. The kinetic energy cutoff was set to 700 eV. Electronic exchange and correlation were described using the functional of Perdew, Burke and Ernzerhof optimized for solids (PBEsol)[23,24] while the pseudopotential approach followed the projector-augmented wave (PAW) method according to Blöchl.[25] Additionally, D3 correction with Becke–Johnson damping[26] was employed to consider van-der-Waals interactions. The Local Orbital Basis Suite Towards Electronic-Structure Reconstruction (LOBSTER)[27,28] was then used to project the optimized wave functions onto a local all-electron Slater-type orbital basis set to gain chemical information, such as wave function-derived atomic charges from Löwdin populations, calculate the COBI, DOS and molecular orbitals.

Thermal Analysis

The thermogravimetric analyses were performed in alumina crucibles, under nitrogen atmosphere and a heating rate of 5 K/min using a STA 409 PC Luxx.

Table S1. Crystal data and structural refinement of $Se_4[S_4O_{13}]$, $Se_4[B(S_2O_7)_2]_2(SO_3)$, $Se_4[B_2(SO_4)_4]$, $Se_4[B_2(SO_4)_2(S_2O_7)_2]$-I, $Se_4[B_2(SO_4)_2(S_2O_7)_2]$-II. Standard deviations are given in parenthesis

	$Se_4[S_4O_{13}]$	$Se_4[B(S_2O_7)_2]_2(SO_3)$	$Se_4[B_2(SO_4)_4]$	$Se_4[B_2(SO_4)_2(S_2O_7)_2]$-I	$Se_4[B_2(SO_4)_2(S_2O_7)_2]$-II
temperature/ K	100(2)	111(2)	100(2)	201(2)	100(2)
molar mass / g·mol^{-1}	652.09	1122.03	721.71	881.82	881.82
crystal system	orthorhombic	monoclinic	monoclinic	monoclinic	monoclinic
crystal size / mm^3	0.10 × 0.09 × 0.02	0.14 × 0.07 × 0.07	0.10 × 0.08 × 0.06	0.13 × 0.11 × 0.09	0.16 × 0.07 × 0.04
space group	$P2_12_12_1$ (No. 19)	$C2/c$ (No. 15)	$C2/c$ (No. 15)	$P2_1/c$ (No.14)	$P2_1/c$ (No.14)
a / Å	12.0942(7)	15.8233(6)	13.376(2)	6.6199(8)	8.2780(3)
b / Å	13.1972(8)	10.2564(4)	7.435(1)	16.603(2)	24.065(1)
c / Å	16.504(1)	17.1486(8)	14.937(3)	9.495(1)	10.5750(4)
β / °	–	105.491(2)	99.277(9)	98.812(5)	105.891(1)
volume / Å^3	2634.3(3)	2681.9(2)	1466.0(4)	1031.3(2)	2026.2(1)
Z	8	4	2	2	2
ρ_{calc} / g·cm^3	3.29	2.78	3.27	2.84	2.89
absorption coefficient μ / mm^{-1}	11.8	6.3	10.7	7.8	7.9
F(000) / e	2432	2152	1352	836	1672
Flack parameter	0.425(7)	–	–	–	–
radiation wavelength λ / Å	0.71073	0.71073	0.71073	0.71073	0.71073
absorption correction	multi-scan	multi-scan	multi-scan	multi-scan	multi-scan
transmission factor (min./max.)	0.475 / 0.746	0.621 / 0.746	0.598 / 0.749	0.665 / 0.749	0.549 / 0.746
Index range $h/k/l$	15, –13 | 16, –17 | ±21	±21 | ±13 | ±22	±21 | ±11 | ±24	±9 | ±23 | ±13	–9, 11 | ±23 | ±13
theta range / °	1.976-28.315	2.393-28.286	2.763-34.999	2.453-29.999	2.558-30.009
reflections collected	33481	28407	33867	59900	66407
independent reflections	6511	3342	3215	3007	5905
observed reflections	6059	2986	2696	2823	4897
refined parameters / restraints	380 / 0	209 / 0	119 / 0	154 / 0	307 / 0
R_{int}	0.043	0.051	0.089	0.055	0.072
R_σ	0.033	0.026	0.045	0.021	0.034
R_1 (all data)	0.026	0.029	0.049	0.019	0.039
wR_2 (all data)	0.047	0.056	0.082	0.045	0.059
Goof	1.059	1.067	1.056	1.061	1.037
residual electron density (min./max.) / e$^-$ Å^3	–0.62 / 0.88	–0.49 / 0.48	–0.92 / 1.29	–0.47 / 0.09	–0.63 / 0.61
ICSD number	2405907	2534710	2534707	2534708	2534709

Table S2. Crystallographic and Rietveld refinement data of $Se_4[B(S_2O_7)_2]_2$

	$Se_4[B(S_2O_7)_2]_2$
Cell mass/g mol^{-1}	4167.86
Crystal system	monoclinic
Space group	*C2/c*
Wavelength/ Å	1.54059/1.54443
a / Å	19.1728(2)
b / Å	10.1834(1)
c / Å	14.5287(2)
β / °	116.521(1)
V / Å^3	2538.15(5)
T / K	298
Z	4
refined parameters	94
ρ_{calc} / g cm^{-3}	2.73(1)
R_{wp}/% [a]	2.24
R_p/% [a]	1.55
R_{Bragg}/% [a]	1.75
Goof [a]	2.45
Starting angle meas. / ° 2*θ*	5
Final angle meas. / ° 2*θ*	80
Starting angle used / ° 2*θ*	5
Final angle used / ° 2*θ*	80
Step width /° 2*θ*	0.02
Time / h	15
ICSD number	2534711

[a]as defined in TOPAS 7.0

Table S3. Atomic coordinates of $Se_4[B(S_2O_7)_2]_2$

Atom	Wychoff	*x/a*	*y/b*	*z/c*	*B* [b]
B1	8*f*	-0.4775(16)	-0.756(5)	1.114(2)	2.55(9)
S1	8*f*	-0.5083(4)	-0.7196(8)	0.9208(6)	2.55(9)
S2	8*f*	-0.6226(4)	-0.7216(8)	0.9806(5)	2.55(9)
S3	8*f*	-0.3657(5)	-0.8827(10)	1.2528(7)	2.55(9)
S4	8*f*	-0.3563(6)	-0.6187(10)	1.2338(7)	2.55(9)
O1	8*f*	-0.5273(9)	-0.8189(14)	0.8444(11)	2.55(9)
O2	8*f*	-0.4622(8)	-0.6193(14)	0.9084(11)	2.55(9)
O3	8*f*	-0.4653(7)	-0.7838(15)	1.0235(11)	2.55(9)
O4	8*f*	-0.5883(9)	-0.6517(13)	0.9147(12)	2.55(9)
O5	8*f*	-0.6544(9)	-0.8500(15)	0.9375(11)	2.55(9)
O6	8*f*	-0.6793(9)	-0.6407(13)	0.9865(13)	2.55(9)
O7	8*f*	-0.5578(8)	-0.740(2)	1.0858(10)	2.55(9)
O8	8*f*	-0.3565(9)	-0.9809(14)	1.3312(13)	2.55(9)
O9	8*f*	-0.3221(9)	-0.9194(18)	1.1959(12)	2.55(9)
O10	8*f*	-0.4480(13)	-0.8679(17)	1.1833(16)	2.55(9)
O11	8*f*	-0.3346(7)	-0.746(2)	1.3067(9)	2.55(9)
O12	8*f*	-0.3428(10)	-0.5004(14)	1.2964(12)	2.55(9)
O13	8*f*	-0.3114(10)	-0.6172(14)	1.1802(12)	2.55(9)
O14	8*f*	-0.4346(12)	-0.6269(17)	1.1655(14)	2.55(9)
Se1	8*f*	0.2606(2)	-0.0938(2)	0.9896(3)	1.42(6)
Se2	8*f*	0.18333(14)	-0.2557(4)	0.88137(17)	1.42(6)

[b] Debye-Waller factors are constrained to be identical within the Se_4 and $[B(S_2O_7)_2]$ groups.

Table S4. Details on Rietveld refinement of $Se_4[S_4O_{13}]$, $Se_4[B(S_2O_7)_2]_2(SO_3)$, $Se_4[B(S_2O_7)_2]_2$, $Se_4[B_2(SO_4)_4]$, $Se_4[B_2(SO_4)_2(S_2O_7)_2]$-I, $Se_4[B_2(SO_4)_2(S_2O_7)_2]$-II (corresponding standard deviations given in parentheses)

	$Se_4[S_4O_{13}]$	$Se_4[B(S_2O_7)_2]_2(SO_3)$	$Se_4[B_2(SO_4)_4]$	$Se_4[B_2(SO_4)_2(S_2O_7)_2]$-I	$Se_4[B_2(SO_4)_2(S_2O_7)_2]$-II
Crystal system	orthorhombic	monoclinic	monoclinic	monoclinic	monoclinic
Space group	$P2_12_12_1$ (no. 19)	$C2/c$ (no. 15)	$C2/c$ (no. 15)	$P2_1/c$ (no. 14)	$P2_1/c$ (no. 14)
a / Å	12.115(1)	15.937(5)	13.486(2)	6.654(1)	8.296(3)
b / Å	13.287(1)	10.379(4)	7.4442(9)	16.629(2)	24.177(6)
c / Å	16.730(3)	17.295(6)	15.014(2)	9.508(2)	10.665(3)
V / Å^3	2693.3(6)	2758.0(2)	1486.3(3)	1040.1(3)	2057.2(5)
β / °	–	105.4(3)	99.57(8)	98.6(1)	1055.9(2)
R_{Bragg}	0.008	0.028	0.023	0.027	0.036
R_{wp}	0.017	0.041	0.027	0.033	0.045

Table S5. Selected bond lengths (in pm) and angles (in °) of $Se_4[S_4O_{13}]$, $Se_4[B(S_2O_7)_2]_2(SO_3)$, $Se_4[B_2(SO_4)_4]$, $Se_4[B_2(SO_4)_4]$, $Se_4[B_2(SO_4)_2(S_2O_7)_2]$-I and $Se_4[B_2(SO_4)_2(S_2O_7)_2]$-II. Standard deviations are given in parentheses

	$Se_4[S_4O_{13}]$	$Se_4[B(S_2O_7)_2]_2(SO_3)$	$Se_4[B_2(SO_4)_4]$	$Se_4[B_2(SO_4)_2(S_2O_7)_2]$-I	$Se_4[B_2(SO_4)_2(S_2O_7)_2]$-II
Se – O_{term}	271.1(3)-371.0(4)	271.9(2)-367.7(2)	272.0(2)-364.7(2)	274.0(1)-363.4(1)	272.9(2)-373.7(3)
Σ r_{ion} Se – O [1]			280		
S – O_{br}	153.6(4)-177.8(4)	151.9(2)-153.8(2)	152.6(2)-155.8(2)	152.7(1)-163.7(1)	152.1(2)-163.7(2)
S – O_{term}	140.9(4)-144.2(4)	140.4(2)-142.7(2)	141.5(2)-143.5(3)	140.8(1)-143.4(1)	140.5(3)-143.0(2)
Σ r_{ion} S – O			147		
B – O	–	146.4(4)-147.5(4)	145.1(3)-149.0(3)	145.0(2)-148.7(2)	144.2(3)-149.9(4)
Σ r_{ion} B – O			146		
O-S-O	96.4(2)-122.2(2)	100.5(1)-122.2(1)	104.32(1)-116.3(1)	100.8(7)-121.3(9)	100.3(1)-121.3(1)
O-B-O	–	105.8(2)-112.3(2)	107.8(3)-113.8(3)	104.1(1)-115.2(1)	106.9(2)-112.6(2)

Table S6. Selected bond lengths (in pm), bond angles (in °), torsion angles (in °) and points groups of the molecular Se_4^{2+} cations in $Se_4[S_4O_{13}]$, $Se_4[B(S_2O_7)_2]_2(SO_3)$, $Se_4[B(S_2O_7)_2]_2$, $Se_4[B_2(SO_4)_4]$, $Se_4[B_2(SO_4)_2(S_2O_7)_2]$-I and $Se_4[B_2(SO_4)_2(S_2O_7)_2]$-II

	$Se_4[S_4O_{13}]$ (1)	$Se_4[S_4O_{13}]$ (2)	$Se_4[B(S_2O_7)_2]_2(SO_3)$	$Se_4[B(S_2O_7)_2]_2$[a]	$Se_4[B_2(SO_4)_4]$	$Se_4[B_2(SO_4)_2(S_2O_7)_2]$-I	$Se_4[B_2(SO_4)_2(S_2O_7)_2]$-II
Se-Se distance / pm	228.53(7)	229.08(7)	228.48(5)	228.6(33)	227.90(5)	228.11(5)	227.58(7)
Se-Se distance / pm	228.63(7)	229.03(8)	228.59(4)	228.2(32)	228.00(4)	228.88(3)	227.74(3)
Se-Se distance / pm	228.75(7)	228.97(7)	–	–	–	–	228.18(3)
Se-Se distance / pm	229.18(6)	228.96(7)	–	–	–	–	228.72(7)
Bond angle / °	90.07(2)	90.15(2)	89.39(1)	90(12)	89.04(2)	89.967(8)	89.32(1)
Bond angle / °	89.98(2)	89.83(3)	90.61(1)	90(13)	90.96(1)	90.03(1)	90.39(2)
Bond angle / °	90.15(2)	90.16(2)	–	–	–	–	89.72(2)
Bond angle / °	89.78(2)	89.85(3)	–	–	–	–	90.57(2)
Torsion angle / °	1(2)	1(2)	0	0	0	0	1(2)
Point group	C_1	C_1	D_{2h}	D_{2h}	D_{2h}	D_{2h}	C_1

[a] the large esds are the result of the refinement from powder data

Table S7. Bond lengths (in pm) of $(S_4O_{13})^{2-}$ groups in $Se_4[S_4O_{13}]$, $(NO_2)_2[S_4O_{13}]$, $[IBr_2]_2[S_4O_{13}]$, $Li_2[S_4O_{13}]$. Standard deviations are given in parentheses

	$Se_4[S_4O_{13}]$[a]	$(NO_2)_2[S_4O_{13}]$[b]	$[IBr_2]_2[S_4O_{13}]$	$Li_2[S_4O_{13}]$
S1-O11	141.7(4)	142.0(9)	144.2(3)	143.1(2)
S1-O12	144.1(3)	142.3(10)	143.3(3)	142.5(2)
S1-O13	144.0(4)	141.6(10)	142.6(3)	142.4(2)
S1-O121	175.6(4)	176.6(9)	171.3(3)	176.5(2)
S2-O121	154.6(4)	153.0(8)	156.3(3)	152.4(2)
S2-O21	140.9(4)	141.4(10)	141.0(3)	140.9(2)
S2-O22	141.2(4)	141.1(9)	141.2(3)	141.0(2)
S2-O231	162.0(4)	163.9(9)	161.6(3)	162.8(2)
S3-O231	163.8(4)	160.9(8)	163.2(3)	161.7(2)
S3-O31	141.0(4)	141.3(9)	140.4(3)	141.7(2)
S3-O32	141.3(4)	142.0(10)	140.0(3)	140.9(2)
S3-O341	154.1(4)	153.4(9)	155.6(3)	153.2(2)
S4-O341	176.3(4)	177.0(9)	170.5(3)	175.3(2)
S4-O41	141.1(4)	142.1(10)	143.7(3)	142.1(2)
S4-O42	143.8(3)	143.1(11)	142.5(3)	141.8(2)
S4-O43	143.0(3)	143.1(10)	144.2(3)	142.1(2)

[a] + [b] Average values for two crystallographic different $[S_4O_{13}]^{2-}$ units in $Se_4[S_4O_{13}]$ and $(NO_2)_2[S_4O_{13}]$, respectively.

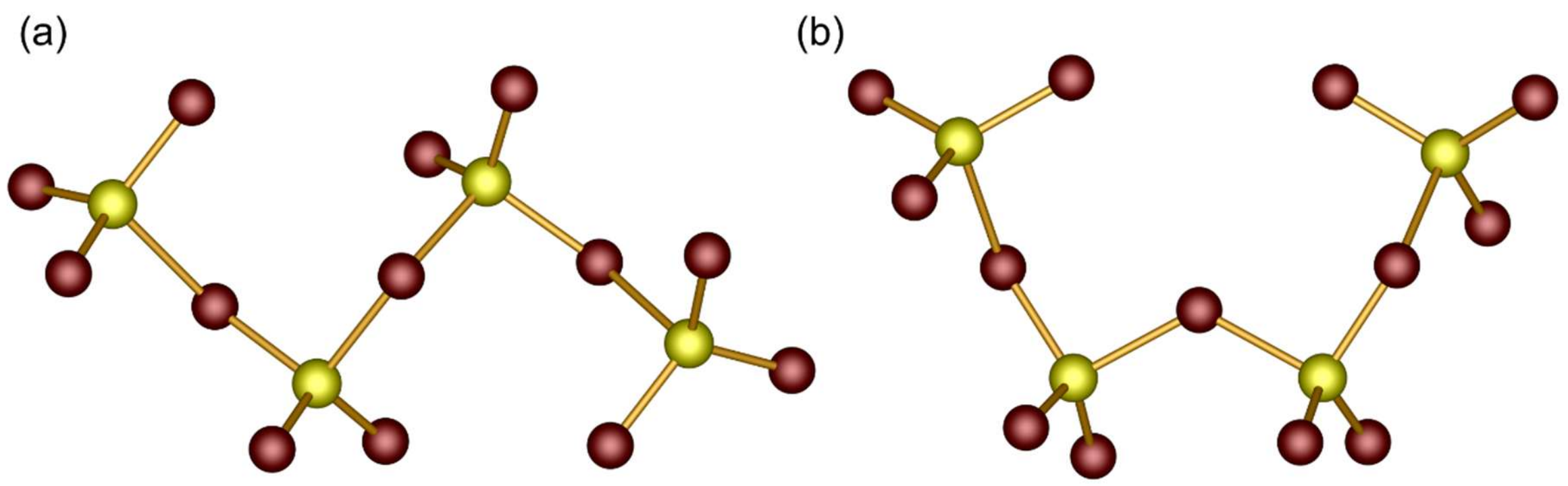

Figure S1. Comparison of the zig-zag and horseshoe-type arrangement of the $S_4O_{13}^{2-}$ units in (a) $Se_4[S_4O_{13}]$ and (b) $(NO_2)_2[S_4O_{13}]$; oxygen is displayed as red spheres, sulfur in yellow.

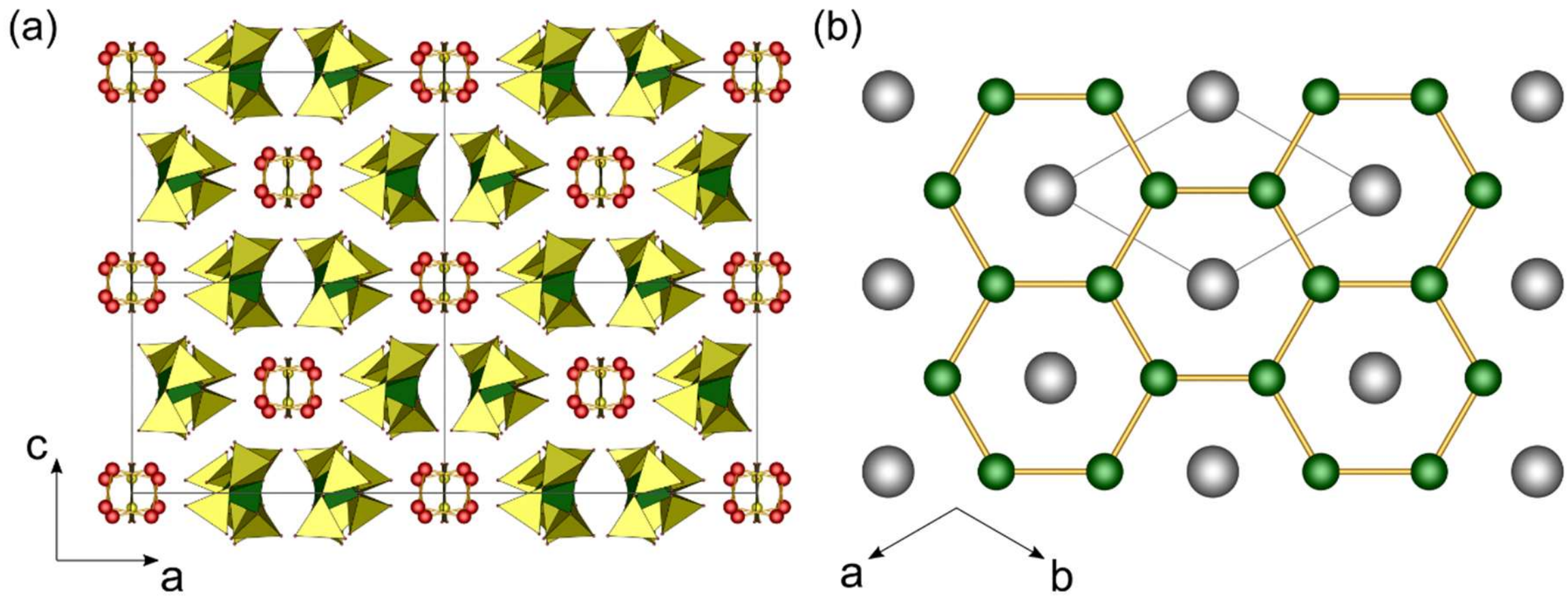

Figure S2. Unit cells of (a) $Se_4[B(S_2O_7)_2]_2(SO_3)$ in comparison to the structurally related (b) AlB_2-type. The $[B(S_2O_7)_2]^{2-}$ unit resembles the position of boron atoms while the Se_4^{2+} rings can be substituted by the aluminium atoms. Aluminium in grey; the boron honeycomb network in green; sulfur and sulfate tetrahedra in yellow and Se_4^{2+} rings in red.

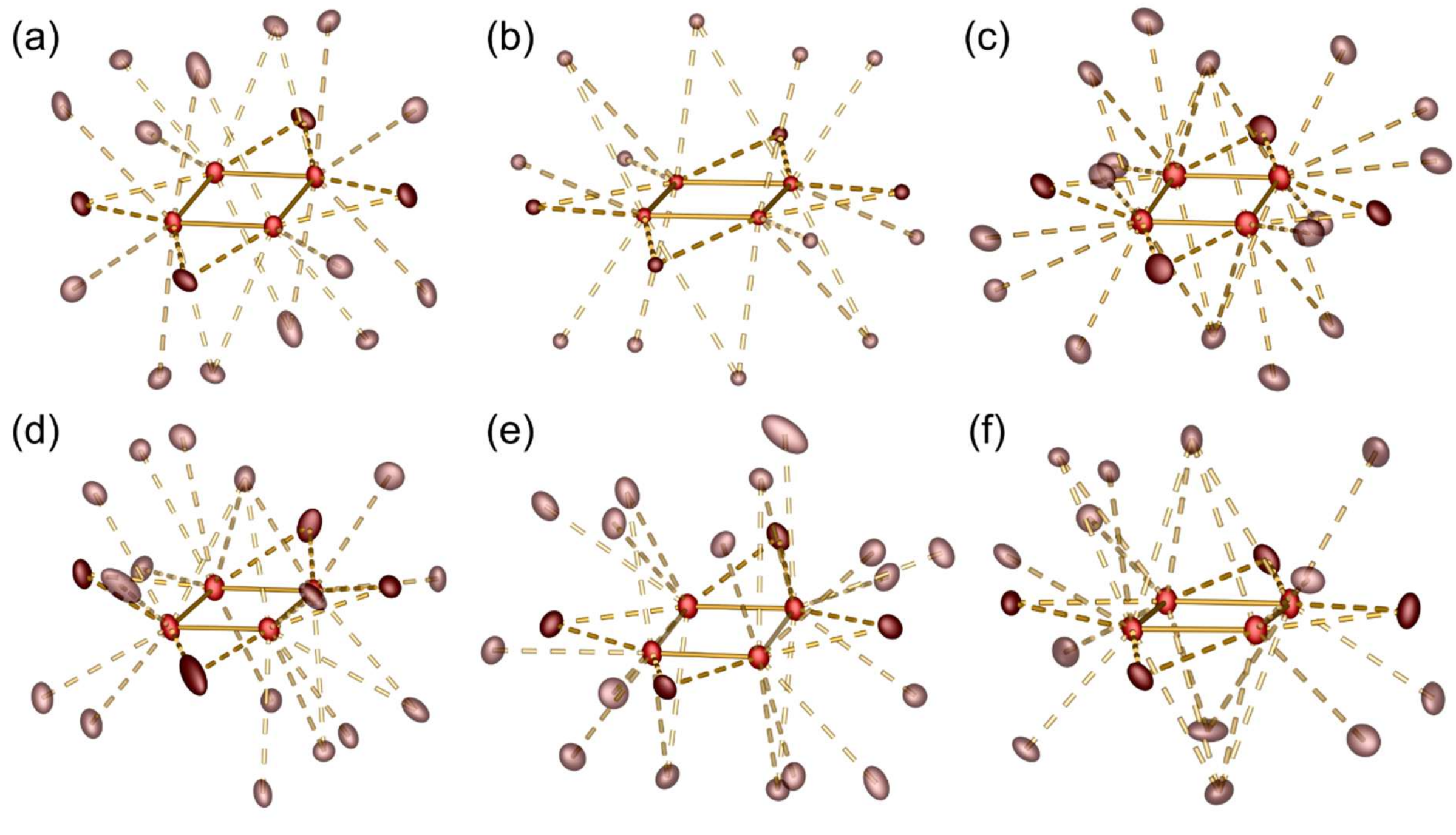

Figure S3. Coordination environment of Se_4^{2+} in (a) $Se_4[B(S_2O_7)_2]_2(SO_3)$, (b) $Se_4[B(S_2O_7)_2]_2$, (c) $Se_4[B_2(SO_4)_2(S_2O_7)_2]$-I (d) $Se_4[S_4O_{13}]$ (1), (e) $Se_4[S_4O_{13}]$ (2) and (f) $Se_4[B_2(SO_4)_2(S_2O_7)_2]$-II. The oxygen atoms coordinating the edges of the ring are highlighted. Oxygen atoms in dark red; selenium in red. Displacement ellipsoids are set to 70% probability except for (b) $Se_4[B(S_2O_7)_2]_2$ which was refined by powder X-ray diffraction data.

Table S8. ECon derived by MAPLE calculations for Se1 and Se2 in $Se_4[B_2(SO_4)_4]$

Atom	*x*	*y*	*z*	Distance / pm	ECon(1)	ECon(9)
**Central atom 1**						
Se1	0.2016	0.4245	0.5426			
O11	0.3467	0.6871	0.5849	275.4	1.298	1.632
O12	-0.0013	0.3427	0.5572	282.4	1.15	1.501
O22	0.2785	0.186	0.7283	331.3	0.289	0.577
O12	0.0013	0.6573	0.4428	333.3	0.267	0.547
O11	0.1533	0.8129	0.4151	346.2	0.147	0.362
O21	0.1487	0.84	0.6303	347.2	0.139	0.349
O24	0.0513	0.6201	0.6899	352.4	0.106	0.289
O23	0.4086	0.401	0.7252	356.4	0.084	0.246
**Next Ligand**	:					
O13	0.0767	0.6003	0.3073	386.8	0.009	0.054
**Central atom 2**						
Se2	0.3549	0.3548	0.5039			
O12	0.5013	0.1573	0.4428	271.9	1.249	1.378
O11	0.3467	0.6871	0.5849	276.1	1.162	1.293
O21	0.3513	0.66	0.3697	302.3	0.629	0.756
O23	0.4086	0.401	0.7252	328.6	0.242	0.328
O14	0.4864	0.3798	0.3288	338.3	0.153	0.22
**Next Ligand**						
014	0.5136	0.6202	0.6712	359.6	0.043	0.072

Table S9. ECon derived by MAPLE calculations for Se1 and Se2 in $Se_4[B(S_2O_7)_2]_2(SO_3)$

Atom	*x*	*y*	*z*	Distance / pm	ECon(1)	ECon(7)
**Central atom 1**						
Se1	0.4153	0.5667	0.518			
O9	0.2613	0.4504	0.4413	271.7	1.374	1.625
O12	0.4777	0.7964	0.6228	296.7	0.854	1.136
O13	0.3179	0.7832	0.5908	314.5	0.527	0.789
O6	0.445	0.8501	0.4442	325.4	0.363	0.596
O16	0.5077	0.3621	0.6809	348.7	0.129	0.273
O1	0.3288	0.439	0.6794	364.3	0.052	0.137
**Next Ligand**						
O5	0.3289	0.7579	0.3323	367.8	0.041	0.115
**Central atom 2**						
Se2	0.5622	0.6118	0.5533			
O12	0.4777	0.7964	0.6228	276.5	1.31	1.409
O9	0.7388	0.5496	0.5587	284.4	1.145	1.248
O8	0.6517	0.7888	0.4556	306.2	0.705	0.807
O2	0.6967	0.8048	0.6609	312.3	0.597	0.694
O6	0.445	0.8501	0.4442	332.5	0.298	0.371
O16	0.5077	0.3621	0.6809	361.9	0.069	0.1
**Next Ligand**						
O15	0.5	0.5674	0.75	378.5	0.022	0.036

Table S10. ECon derived by MAPLE calculations for Se1 and Se2 in $Se_4[B(SO_4)_2(S_2O_7)_2]_2$-I

Atom	*x*	*y*	*z*	Distance / pm	ECon(1)	ECon(5)
**Central atom 1**						
Se1	0.8788	0.4244	0.0545			
O12	0.5499	0.4745	0.1751	274.0	1.256	1.373
O22	1.0528	0.2779	-0.0049	278.7	1.156	1.276
O31	0.7342	0.2878	0.2275	304.1	0.643	0.759
O32	0.5472	0.3167	-0.1608	328.9	0.269	0.351
O11	1.2613	0.5014	0.3011	342.2	0.145	0.203
O11	0.7387	0.4986	-0.3011	357.9	0.058	0.091
**Next Ligand**						
O12	0.4501	0.5255	-0.1751	370.4	0.024	0.042
**Central atom 2**						
Se2	0.8505	0.5611	0.0672			
O12	0.5499	0.4745	0.1751	277.4	1.298	1.478
O22	0.9472	0.7221	0.005	283.3	1.177	1.363
O21	0.8991	0.6756	0.3162	301.2	0.811	1.003
O12	0.4501	0.5255	-0.1751	328.6	0.353	0.505
O11	1.2613	0.5014	0.3011	338.5	0.238	0.365
O32	0.4528	0.6833	0.1608	354.3	0.11	0.194
O11	0.7387	0.4986	-0.3011	361.4	0.074	0.138
O4	0.6252	0.7289	-0.1473	363.4	0.065	0.125
O13	0.6047	0.5517	0.3885	367.4	0.051	0.102
**Next Ligand**						
O32	1.4528	0.6833	0.1608	444.1	0.000	0.000

Table S11. ECon derived by MAPLE calculations for Se1, Se2, Se3 and Se4 in $Se_4[B(SO_4)_2(S_2O_7)_2]_2$-II

Atom	*x*	*y*	*z*	Distance / pm	ECon(1)	ECon(7)
**Central atom 1**						
Se1	0.1469	0.4245	0.1203			
O61	-0.1722	0.3951	-0.0194	274.3	1.251	1.352
O62	0.3182	0.5248	0.1416	277.8	1.175	1.278
O21	-0.0699	0.5148	0.1461	287.9	0.961	1.067
O21	0.0699	0.4852	-0.1461	308.1	0.572	0.668
O54	0.0461	0.3559	-0.17	338.3	0.177	0.232
O12	0.1537	0.4283	0.4483	345.5	0.122	0.167
O51	0.3112	0.3201	-0.0468	354.9	0.071	0.102
O23	0.3289	0.4412	-0.1495	359.5	0.053	0.079
Next Ligand	:					
O22	-0.2938	0.4601	0.1965	403.8	0.001	0.002
**Central atom 2**						
Se2	0.0771	0.3363	0.1627			
O52	0.1462	0.2304	0.259	274.7	1.248	1.408
O61	-0.1722	0.3951	-0.0194	279.2	1.153	1.317
O41	-0.0424	0.3115	0.4125	312.2	0.509	0.66
O51	0.3112	0.3201	-0.0468	334.2	0.218	0.323
O54	0.0461	0.3559	-0.17	348.9	0.103	0.172
O434	-0.2265	0.2298	0.0255	360.5	0.051	0.094
Next Ligand	:					
O12	0.1537	0.4283	0.4483	365.7	0.036	0.07
**Central atom 3**						
Se3	0.3424	0.3232	0.2944			
O52	0.1462	0.2304	0.259	272.8	1.309	1.474
O42	0.6875	0.3565	0.401	288.2	0.983	1.16
O32	0.525	0.3053	0.5868	307.8	0.602	0.77
O31	0.6585	0.2834	0.1724	335.8	0.213	0.323
O51	0.3112	0.3201	-0.0468	354.7	0.079	0.141
O12	0.1537	0.4283	0.4483	358.8	0.062	0.115
Next Ligand	:					
O41	-0.0424	0.3115	0.4125	373.7	0.022	0.048
**Central atom 4**						
Se4	0.4141	0.4114	0.2496			
O42	0.6875	0.3565	0.401	272.9	1.297	1.4
O22	0.7062	0.4601	0.1965	287.7	0.983	1.093
O62	0.3182	0.5248	0.1416	298.2	0.77	0.878
O11	0.6883	0.4858	0.4773	334.1	0.224	0.291
O12	0.1537	0.4283	0.4483	342.2	0.153	0.206
Ligand	:					
O51	0.3112	0.3201	-0.0468	373.0	0.022	0.036

Table 12. ECon derived by MAPLE calculations for Se1, Se2, Se3 and Se4 in $Se_4[S_4O_{13}]$

Atom	*x*	*y*	*z*	Distance / pm	ECon(1)	ECon(7)
**Central atom 1**						
Se1	0.7125	0.5842	0.8345			
O11	0.8732	0.7281	0.8137	273.8	1.351	1.533
O43	0.4879	0.6354	0.8517	281.3	1.196	1.387
O21	0.6545	0.804	0.7835	310.0	0.624	0.814
O81	0.701	0.7466	0.9755	316.7	0.512	0.692
O62	0.762	0.5513	0.6454	320.7	0.448	0.621
O82	0.6074	0.5998	1.0297	346.9	0.151	0.255
O671	0.8266	0.5009	1.017	349.1	0.136	0.233
O71	0.9196	0.6543	0.9845	364.1	0.057	0.115
**Next Ligand**						
O41	0.4405	0.5734	0.7211	378.7	0.02	0.05
**Central atom 2**						
Se2	0.624	0.4337	0.811			
O42	0.6148	0.2415	0.7355	282.8	1.472	1.635
O52	0.5488	0.2554	0.9194	309.3	0.952	1.139
O43	0.4879	0.6354	0.8517	320.1	0.748	0.933
O41	0.4405	0.5734	0.7211	324.4	0.672	0.853
O22	0.3649	0.3852	0.8502	326.3	0.639	0.819
O72	0.5437	0.4243	0.6177	333.7	0.519	0.689
O62	0.762	0.5513	0.6454	355.9	0.238	0.361
O61	0.7158	0.3528	1.0185	375.5	0.094	0.168
**Next Ligand**	:					
O781	0.7171	0.3439	0.5946	392.8	0.033	0.071
**Central atom 3**						
Se3	0.7914	0.3563	0.7917			
O42	0.6148	0.2415	0.7355	277.8	1.256	1.478
O13	1.0176	0.3542	0.7559	279.9	1.213	1.439
O31	0.8281	0.1208	0.7705	315.9	0.513	0.73
O781	0.7171	0.3439	0.5946	337.9	0.223	0.378
O83	0.8825	0.2459	0.6102	350.9	0.118	0.23
O53	0.9227	0.2612	0.9658	351.4	0.115	0.225
O62	0.762	0.5513	0.6454	354.8	0.096	0.194
**Next Ligand**						
O12	1.0422	0.1858	0.8154	379.6	0.018	0.052
**Central atom 4**						
Se4	0.8801	0.5063	0.8171			
O13	1.0176	0.3542	0.7559	279.5	1.393	1.529
O11	0.8732	0.7281	0.8137	292.9	1.121	1.269
O32	1.128	0.5212	0.8522	306.1	0.859	1.009
O62	0.762	0.5513	0.6454	322.8	0.556	0.694
O12	0.9578	0.6858	0.6846	335.9	0.364	0.481
O671	0.8266	0.5009	1.017	336.3	0.358	0.475
O71	0.9196	0.6543	0.9845	341.7	0.292	0.398
**Next Ligand**	:					
O51	1.0421	0.4009	1.0024	388.9	0.021	0.042

Table 13. ECon derived by MAPLE calculations for Se5, Se6, Se7 and Se8 in $Se_4[S_4O_{13}]$

**Atom**	***x***	***y***	***z***	**Distance / pm**	**ECon(1)**	**ECon(7)**
**Central atom 5**						
Se5	0.6264	0.1106	0.4556			
O81	0.799	0.2534	0.4755	283.1	1.293	1.482
O82	0.3926	0.0998	0.4703	284.2	1.271	1.462
O11	0.6268	0.2719	0.3137	316.6	0.636	0.831
O71	0.5804	0.3457	0.4845	318.8	0.597	0.789
O43	0.5121	0.1354	0.6483	348.3	0.207	0.332
O341	0.6927	0.0916	0.6622	351.1	0.182	0.299
O83	0.3825	0.2541	0.3898	367.1	0.08	0.153
O231	0.7465	0.0441	0.258	367.7	0.077	0.148
**Next Ligand**						
O13	0.5176	0.1458	0.2441	376.0	0.046	0.098
**Central atom 6**						
Se6	0.5573	0.9489	0.455			
O53	0.5773	0.7388	0.4658	278.9	1.264	1.446
O82	0.3926	1.0998	0.4703	282.8	1.182	1.368
O12	0.4578	0.8142	0.3154	314.8	0.557	0.736
O22	0.6351	0.8852	0.6498	345.5	0.171	0.278
O31	0.6719	0.8792	0.2705	346.9	0.159	0.262
O52	0.4512	0.7554	0.5806	353.1	0.116	0.202
O61	0.2842	0.8528	0.4815	356.5	0.096	0.173
**Next Ligand**						
O62	0.262	0.9487	0.3546	393.7	0.006	0.018
**Central atom 7**						
Se7	0.7333	0.8863	0.4638			
O53	0.5773	0.7388	0.4658	271.2	1.285	1.479
O51	0.9579	0.9009	0.4976	277.9	1.138	1.34
O31	0.6719	0.8792	0.2705	327.8	0.262	0.407
O22	0.6351	0.8852	0.6498	329.1	0.248	0.388
O52	0.9512	0.7446	0.4194	331.4	0.223	0.357
O61	0.7842	0.6472	0.5185	333.9	0.198	0.325
**Next Ligand**	:					
O121	0.8034	0.8018	0.6816	385.8	0.005	0.017
**Central atom 8**						
Se8	0.8022	0.0476	0.462			
O81	0.799	0.2534	0.4755	272.5	1.257	1.477
O51	0.9579	-0.0991	0.4976	276.4	1.174	1.4
O32	0.872	0.0212	0.6478	319.9	0.361	0.551
O72	1.0437	0.0757	0.3823	322.5	0.327	0.51
O231	0.7465	0.0441	0.258	343.5	0.123	0.236
O21	0.8455	0.196	0.2835	357.6	0.053	0.12
**Next Ligand**						
O341	0.6927	0.0916	0.6622	360.6	0.043	0.103

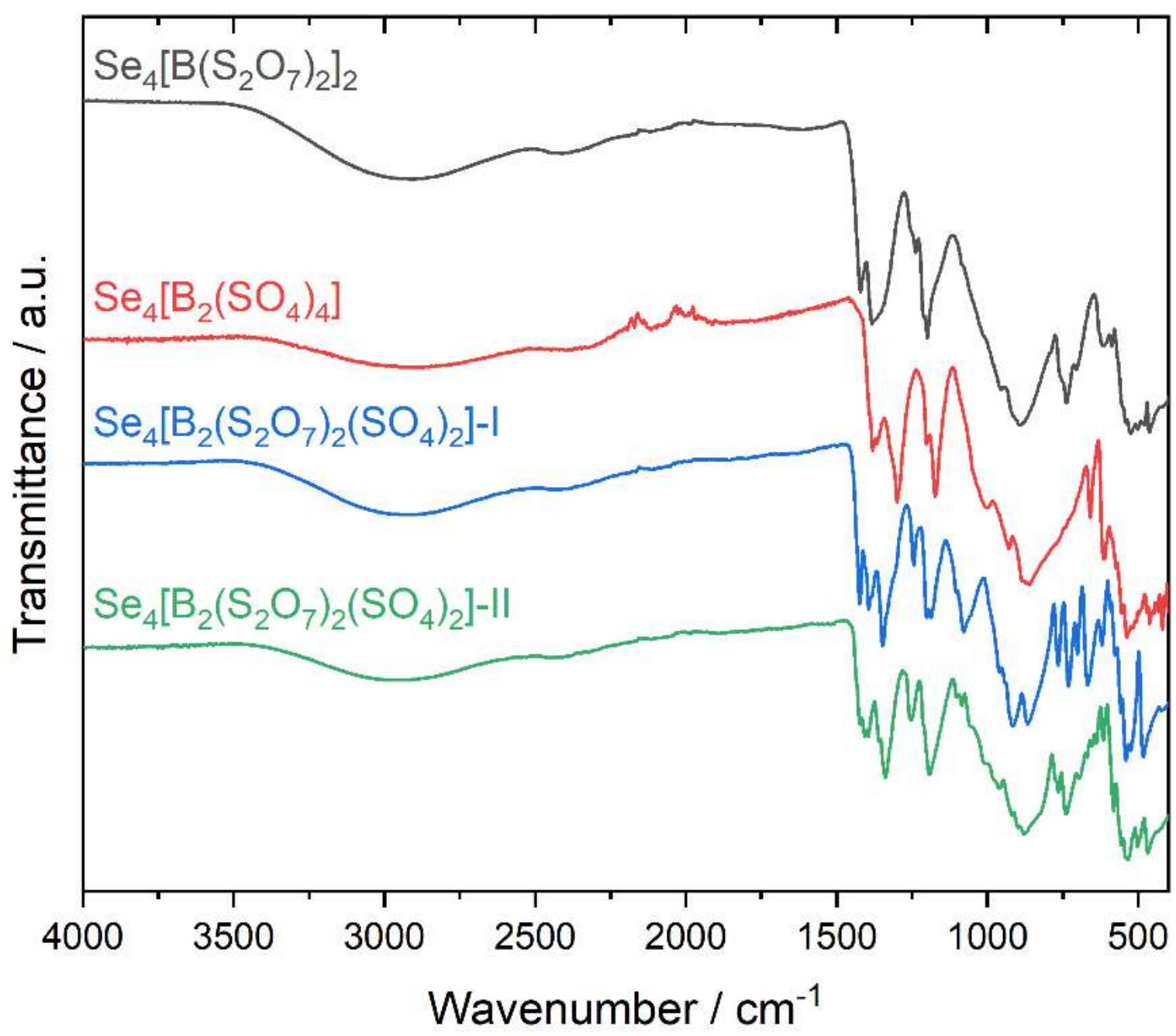

Figure S4. Infrared spectra of $Se_4[B(S_2O_7)_2]_2$, $Se_4[B_2(SO_4)_4]$, $Se_4[B_2(SO_4)_2(S_2O_7)_2]$-I and $Se_4[B_2(SO_4)_2(S_2O_7)_2]$-II between 4000 and 400 cm^{-1}.

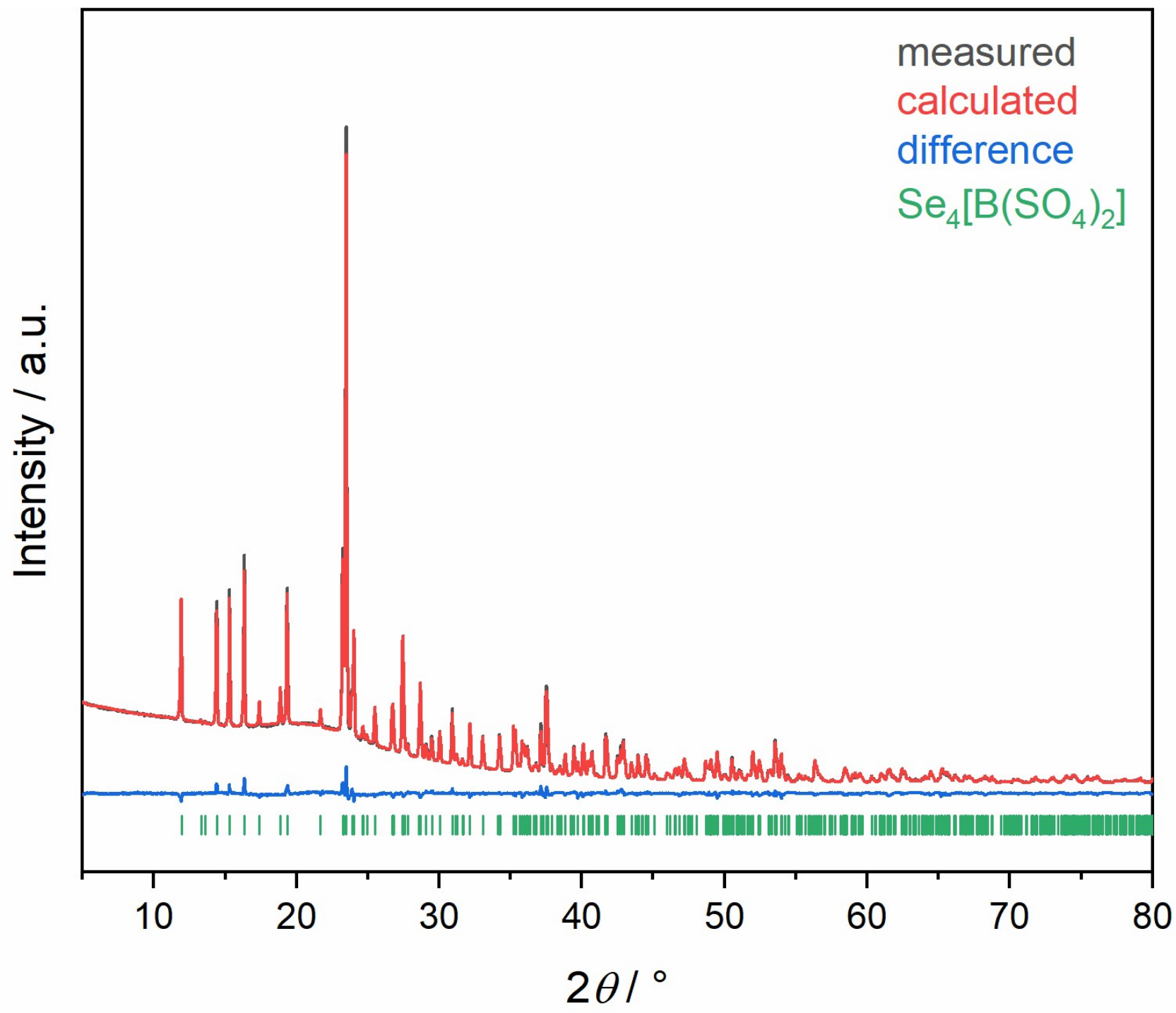

Figure S5. Rietveld refinement of $Se_4[B_2(SO_4)_4]$; measured (grey) and calculated (red) diffraction pattern; difference plot (blue) and theoretical reflection positions (green).

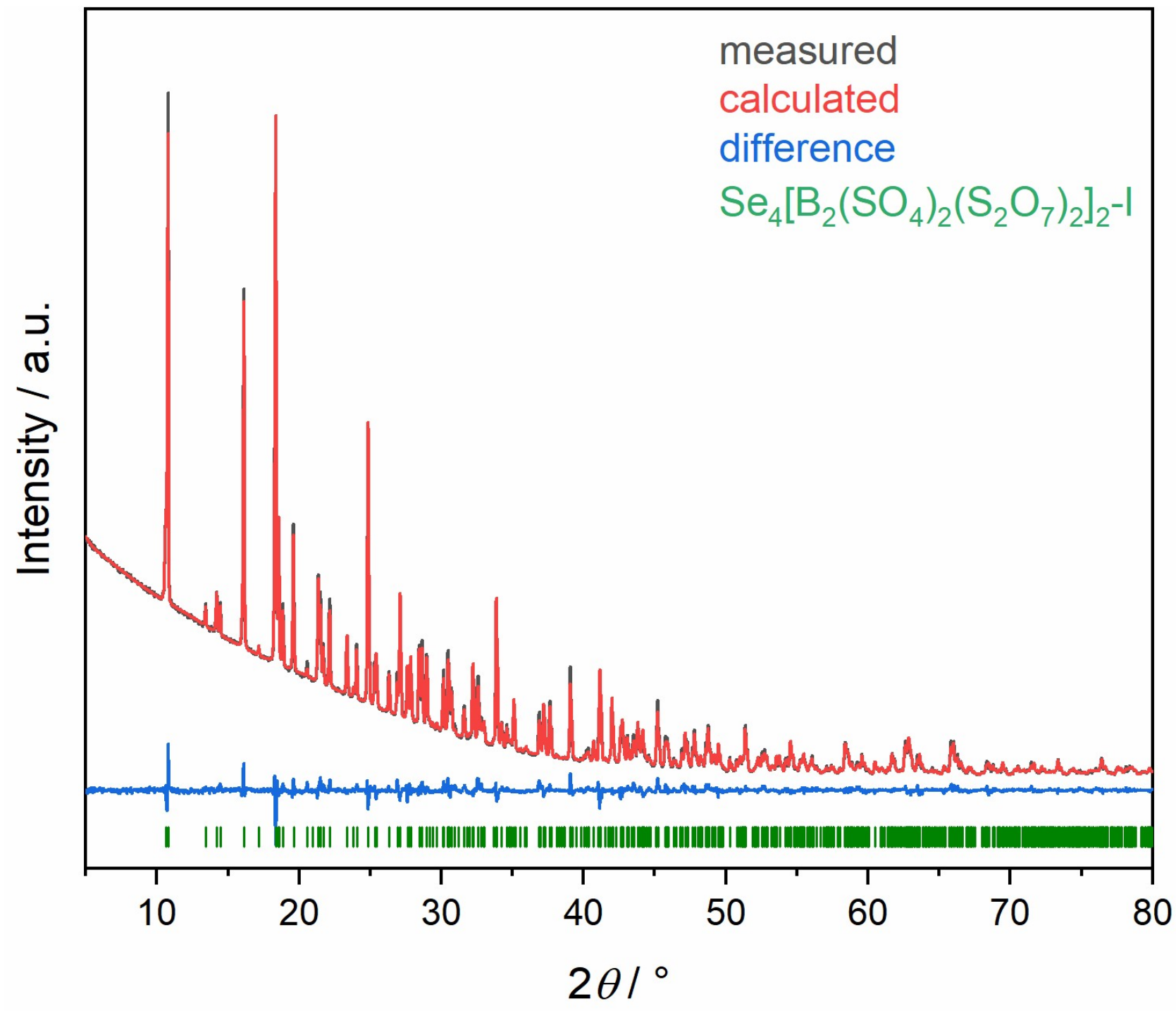

Figure S7. Rietveld refinement of $Se_4[B_2(SO_4)_2(S_2O_7)_2]$-I; measured (grey) and calculated (red) diffraction pattern; difference plot (blue) and theoretical reflection positions (green).

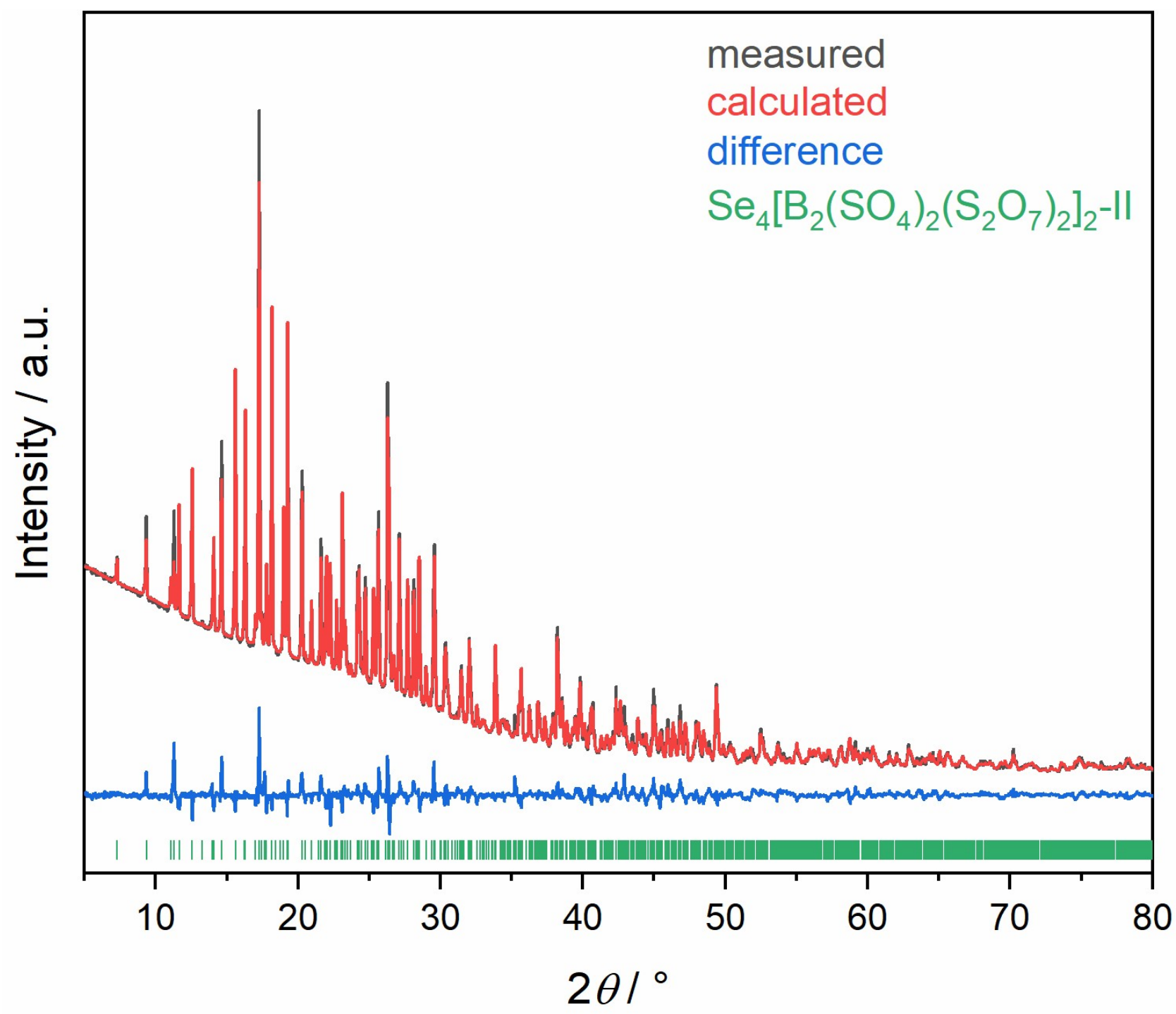

Figure S6. Rietveld refinement of $Se_4[B_2(SO_4)_2(S_2O_7)_2]$-II; measured (grey) and calculated (red) diffraction pattern; difference plot (blue) and theoretical reflection positions (green).

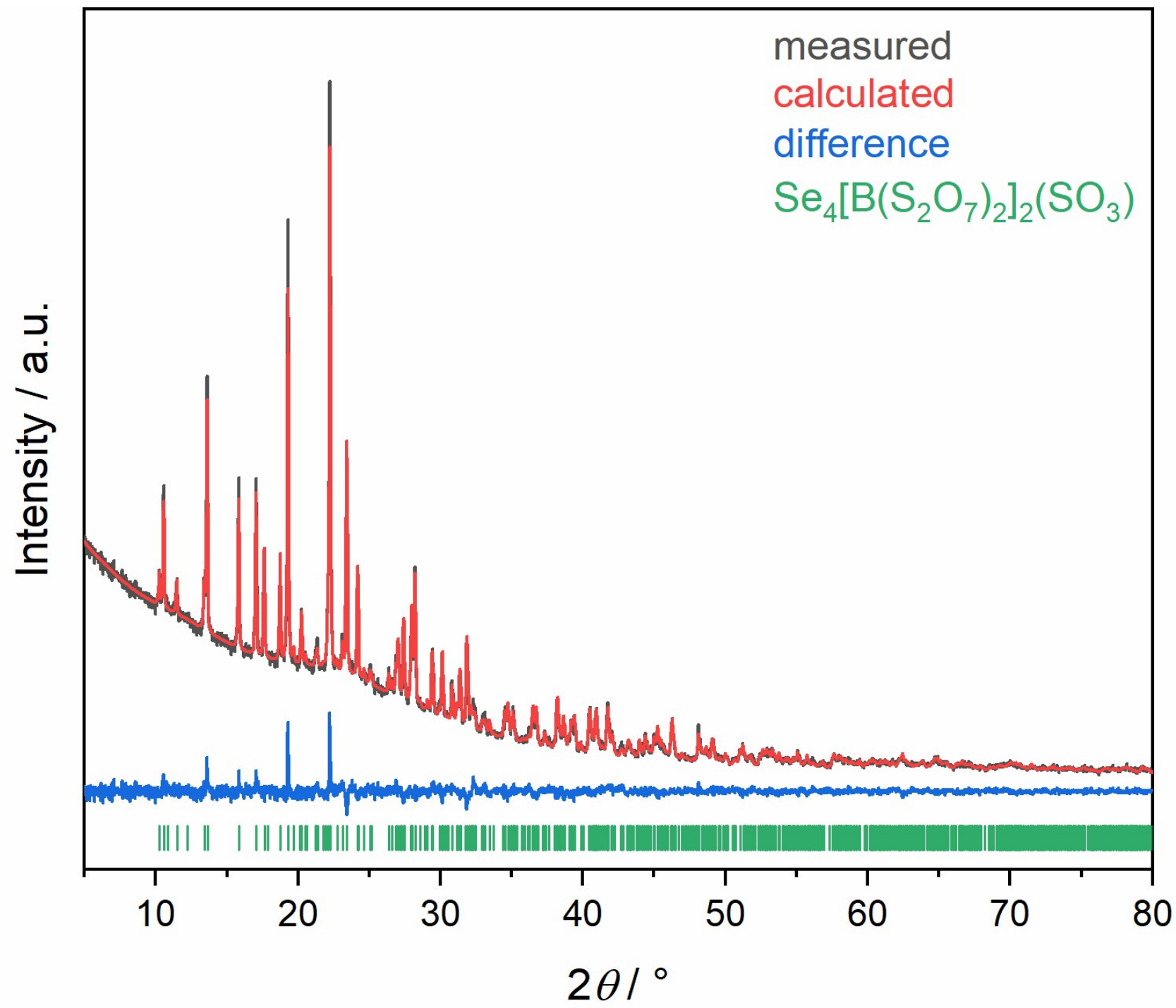

Figure S8. Rietveld refinement of $Se_4[B(S_2O_7)_2]_2(SO_3)$; measured (grey) and calculated (red) diffraction pattern; difference plot (blue) and theoretical reflection positions (green).

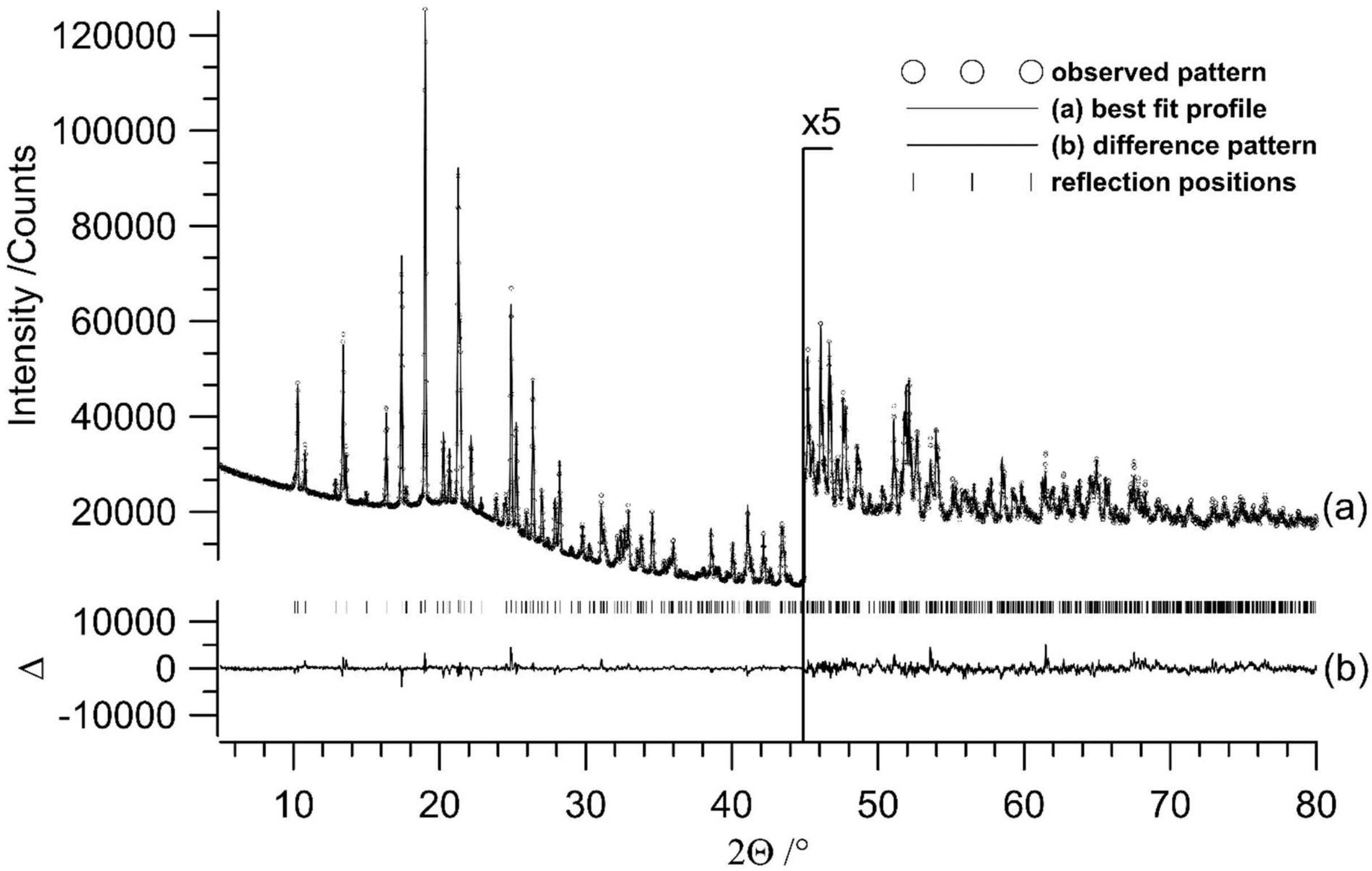

Figure S9. Scattered X-ray (λ = 1.54059/1.54443 Å) diffraction intensities of $Se_4[B(S_2O_7)_2]_2$ at ambient conditions as a function of °2*θ*. The observed pattern (open circles) measured in Debye-Scherrer geometry, the best Rietveld fit profile (solid line) and the difference curves between the observed and the calculated profiles (solid line in separate window below) are shown. The peak positions of the three phases are presented as vertical lines below the powder pattern. The higher angle part is magnified by a factor of 5 for better visibility.

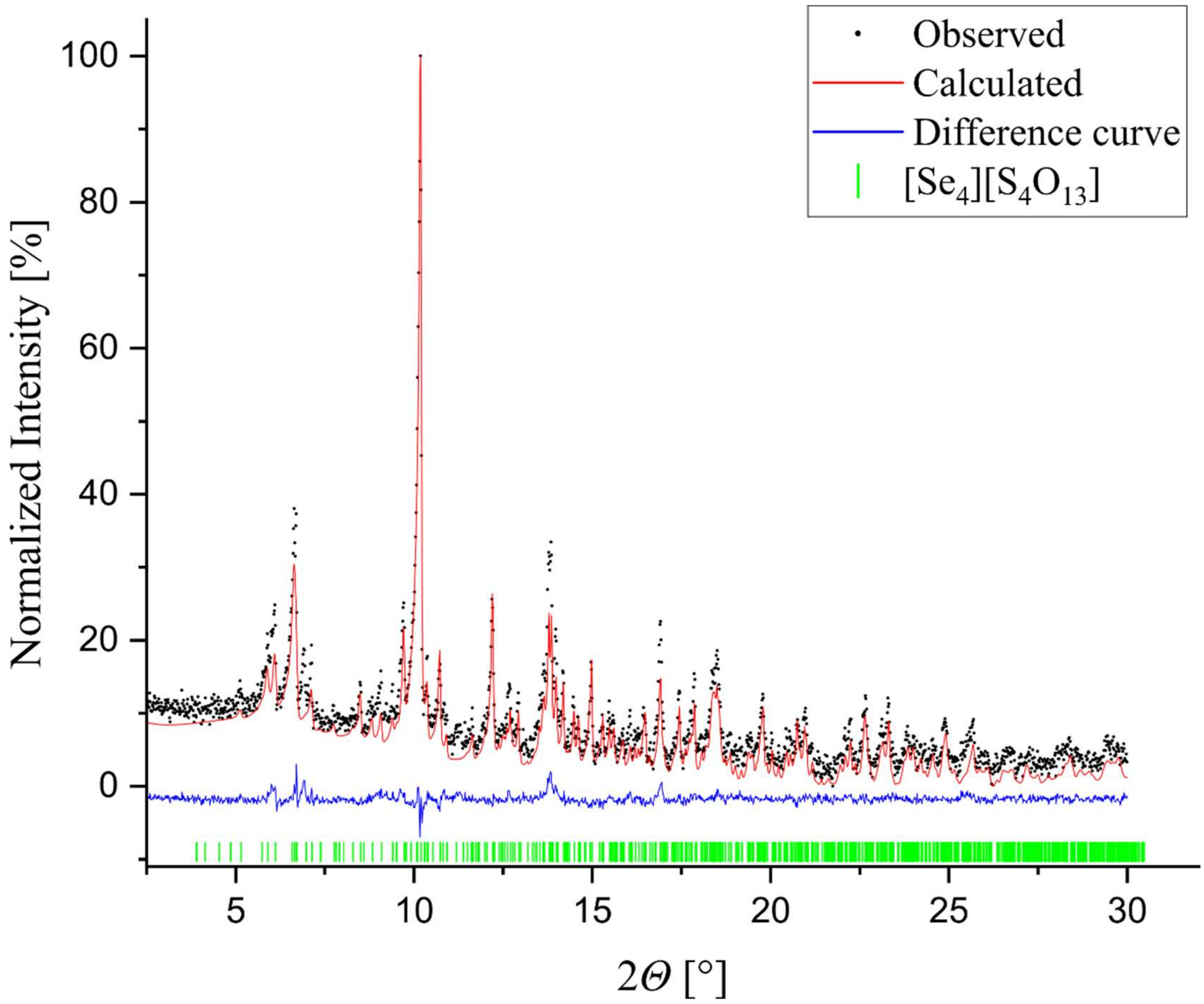

Figure S10. Rietveld refinement of $Se_4[S_4O_{13}]$; measured (black) and calculated (red) diffraction pattern; difference plot (blue) and theoretical reflection positions (green).

References

[1] Bruker, *SADABS*, Bruker AXS Inc., Madison, Wisconsin, USA, **2012**.

[2] G. M. Sheldrick, "A short history of SHELX", *Acta Crystallogr. A* **2008**, *64*, 112.

[3] G. M. Sheldrick, "Crystal structure refinement with SHELXL", *Acta Crystallogr. C* **2015**, *71*, 3.

[4] Bruker, *APEX 5 v2023.9-2*, Bruker AXS Inc., Madison, Wisconsin, USA, **2023**.

[5] L. Krause, R. Herbst-Irmer, G. M. Sheldrick, D. Stalke, "Comparison of silver and molybdenum microfocus X-ray sources for single-crystal structure determination", *J. Appl. Crystallogr.* **2015**, *48*, 3.

[6] Bruker AXS, *Saint V8.40B*, Madison, Wisconsin, USA, **2012**.

[7] O. V. Dolomanov, L. J. Bourhis, R. J. Gildea, J. A. K. Howard, H. Puschmann, "OLEX2 : a complete structure solution, refinement and analysis program", *J. Appl. Crystallogr.* **2009**, *42*, 339.

[8] G. M. Sheldrick, "SHELXT - integrated space-group and crystal-structure determination", *Acta Crystallogr. A* **2015**, *71*, 3.

[9] A. A. Coelho, "TOPAS and TOPAS-Academic: an optimization program integrating computer algebra and crystallographic objects written in C++", *J. Appl. Crystallogr.* **2018**, *51*, 210.

[10] P. Thompson, D. E. Cox, J. B. Hastings, "Rietveld Refinement of Debye-Scherrer Synchrotron X-ray Data from Al_2O_3", *J. Appl. Crystallogr.* **1987**, *20*, 79.

[11] A. A. Coelho, "Indexing of powder diffraction patterns by iterative use of singular value decomposition", *J. Appl. Crystallogr.* **2003**, *36*, 86.

[12] G. S. Pawley, "Unit-Cell Refinement From Powder Diffraction Scans", *J. Appl. Crystallogr.* **1981**, *14*, 357.

[13] R. W. Cheary, A. A. Coelho, J. P. Cline, "Fundamental Parameters Line Profile Fitting in Laboratory Diffractometers", *J. Res. Natl. Inst. Stand. Technol.* **2004**, *109*, 1.

[14] A. A. Coelho, "A charge-flipping algorithm incorporating the tangent formula for solving difficult structures", *Acta Crystallogr. A* **2007**, *63*, 400.

[15] A. A. Coelho, "Whole-profile structure solution from powder diffraction data using simulated annealing", *J. Appl. Crystallogr.* **2000**, *33*, 899.

[16] H. M. Rietveld, "A Profile Refinement Method for Nuclear and Magnetic Structures", *J. Appl. Crystallogr.* **1969**, *2*, 65.

[17] *Origin Pro 2024 (version 10.1.0.170)*, OriginLab Corporation, Northampton, MA (USA), **2024**.

[18] *CorelDRAW Graphics Suite 2017 (version 19.0.0.328)*, Corel Corporation, Ottawa, Ontario (Canada), **2017**.

[19] G. Kresse, J. Hafner, "*Ab initio* molecular dynamics for liquid metals", *Phys. Rev. B* **1993**, *47*, 558.

[20] G. Kresse, J. Furthmüller, "Efficiency of ab-initio total energy calculations for metals and semiconductors using a plane-wave basis set", *Comput. Mat. Sci.* **1996**, *6*, 15.

[21] G. Kresse, J. Furthmüller, "Efficient iterative schemes for ab initio total-energy calculations using a plane-wave basis set", *Phys. Rev. B* **1996**, *54*, 11169.

[22] G. Kresse, D. Joubert, "From ultrasoft pseudopotentials to the projector augmented-wave method", *Phys. Rev. B* **1999**, *59*, 1758.

[23] J. P. Perdew, A. Ruzsinszky, G. I. Csonka, O. A. Vydrov, G. E. Scuseria, L. A. Constantin, X. Zhou, K. Burke, "Restoring the density-gradient expansion for exchange in solids and surfaces", *Phys. Rev. Lett.* **2008**, *100*, 136406.

[24] J. P. Perdew, A. Ruzsinszky, G. I. Csonka, O. A. Vydrov, G. E. Scuseria, L. A. Constantin, X. Zhou, K. Burke, "Erratum: Restoring the Density-Gradient Expansion for Exchange in Solids and Surfaces [Phys. Rev. Lett. 100 , 136406 (2008)]", *Phys. Rev. Lett.* **2009**, *102*.

[25] P. E. Blöchl, "Projector augmented-wave method", *Phys. Rev. B* **1994**, *50*, 17953.

[26] S. Grimme, S. Ehrlich, L. Goerigk, "Effect of the damping function in dispersion corrected density functional theory", *J. Comput. Chem.* **2011**, *32*, 1456.

[27] P. C. Müller, L. S. Reitz, D. Hemker, R. Dronskowski, "Orbital-based bonding analysis in solids", *Chem. Sci.* **2025**, *16*, 12212.

[28] P. C. Müller, C. Ertural, J. Hempelmann, R. Dronskowski, "Crystal Orbital Bond Index: Covalent Bond Orders in Solids", *J. Phys. Chem. C* **2021**, *125*, 7959.
